# Subducting plate structure and megathrust morphology from deep seismic imaging linked to earthquake rupture segmentation at Cascadia

**DOI:** 10.1126/sciadv.adl3198

**Published:** 2024-06-07

**Authors:** Suzanne M. Carbotte, Brian Boston, Shuoshuo Han, Brandon Shuck, Jeffrey Beeson, J. Pablo Canales, Harold Tobin, Nathan Miller, Mladen Nedimovic, Anne Tréhu, Michelle Lee, Madeleine C. Lucas, Hanchao Jian, Danqi Jiang, Liam Moser, Chris Anderson, Darren Judd, Jaime Fernandez, Chuck Campbell, Antara Goswami, Rajendra Gahlawat

**Affiliations:** ^1^ Lamont-Doherty Earth Observatory of Columbia University, Palisades, NY 10964, USA.; ^2^ Auburn University, Auburn, AL 36849, USA.; ^3^ University of Texas at Austin Institute for Geophysics, Austin, TX 78758, USA.; ^4^ Oregon State University, Corvallis, OR 97331, USA.; ^5^ NOAA Pacific Marine Environmental Laboratory (PMEL), Newport, OR 97365, USA.; ^6^ Woods Hole Oceanographic Institution, Falmouth, MA 02543, USA.; ^7^ University of Washington, Seattle, WA 98195, USA.; ^8^ U.S. Geological Survey, Woods Hole Coastal and Marine Science Center, Woods Hole, MA 02543, USA.; ^9^ Dalhousie University, Halifax, NS B3H 4R2, Canada.; ^10^ MIT-WHOI Joint Program in Oceanography/Applied Ocean Science and Engineering, Cambridge and Woods Hole, MA 02139, USA.; ^11^ ION Geophysical, 2105 City West Boulevard, Suite 100, Houston, TX 77042, USA.; ^12^ ACCEL Services Inc., 448 W 19th St., Houston, TX 77008, USA.

## Abstract

The origin of rupture segmentation along subduction zone megathrusts and linkages to the structural evolution of the subduction zone are poorly understood. Here, regional-scale seismic imaging of the Cascadia margin is used to characterize the megathrust spanning ~900 km from Vancouver Island to the California border, across the seismogenic zone to a few tens of kilometers from the coast. Discrete domains in lower plate geometry and sediment underthrusting are identified, not evident in prior regional plate models, which align with changes in lithology and structure of the upper plate and interpreted paleo-rupture patches. Strike-slip faults in the lower plate associated with oblique subduction mark boundaries between regions of distinct lower plate geometry. Their formation may be linked to changes in upper plate structure across long-lived upper plate faults. The Juan de Fuca plate is fragmenting within the seismogenic zone at Cascadia as the young plate bends beneath the heterogeneous upper plate resulting in structural domains that coincide with paleo-rupture segmentation.

## Teaser

A fragmented lower plate in the Cascadia seismogenic zone bounds structural domains linked to paleo-rupture segmentation.

## INTRODUCTION

Subduction zone megathrust faults rupture in patches and repeat ruptures can have similar slip distributions (*1*, *2*). However, dynamic rupture processes are complex and slip histories show that rupture segments can be maintained in some events and breached in others. What gives rise to rupture segmentation and its persistence over repeat earthquake cycles is a question of ongoing debate and a critical need for hazard assessment and monitoring [e.g., (*3*, *4*)]. The geometry of the plate interface plays an important role in modulating rupture characteristics and size [e.g., (*5*–*7*)] and there is considerable evidence that morphologic heterogeneities on the lower plate including seamounts and fracture zones can serve as barriers to rupture propagation in some settings (*2*, *8*). The geometry and depth of the plate boundary zone also contributes to other fault zone properties fundamental for seismogenesis and earthquake propagation, including depth-dependent frictional properties and rheology, and the mineral alteration and dehydration reactions that contribute to the hydrogeology of the plate interface fault (*9*, *10*). The morphology and depth of the plate interface evolves as the downgoing lithospheric plate bends in response to regional tectonic stresses and as a function of its strength and structure. Plate bending typically begins in the outer rise and can result in spatial variations in brittle deformation and near-trench plate hydration that leads to weakening of the subducting plate (*11*) and contributes to the distribution of seismicity further downdip (*12*). Recent studies highlight how variations in bending of the lower plate can develop under the wedge due to along-margin changes in the strength and weight of the upper plate, also contributing to segmentation in earthquake slip along the plate interface (*13*, *14*). Other studies indicate variations in upper plate strength and lithology modulate frictional state along the plate interface and may play the primary role in rupture segmentation (*15*–*17*). Unraveling the role of upper and lower plate properties and their impact on fault frictional state and rupture potential is complex and requires detailed characterization of plate interface geometry and physical properties across multiple rupture segments. This information is currently lacking at most subduction zones.

The Cascadia subduction zone (CSZ) has hosted giant earthquakes of moment magnitude >8.5 in the past and poses a major geohazard to populations of the Pacific Northwest (*7*). The CSZ exhibits along-margin segmentation in paleoslip indicators, current state of locking, plate interface microseismicity, and in patterns of episodic tremor and slip (ETS) (*17*–*23*). However, the extent to which indicators of paleo-rupture segmentation at Cascadia reflect the presence of persistent barriers to rupture propagation, how variations in current fault locking are linked to paleo-rupture segmentation, and what physical characteristics are driving megathrust segmentation are poorly understood as is the significance of these observations for dynamic rupture in future earthquakes [e.g., (*3**,* *4*)]. Accurate characterization of plate interface depth and geometry is needed to evaluate earthquake source and rupture scenarios but with the unusual lack of background seismicity within the seismogenic zone along much of this margin (*20*, *24*, *25*), its geometry and depth have greater uncertainty than at many subduction zones. Data constraints for the seismogenic zone, which is inferred to lie largely offshore at Cascadia (*4*, *9*) are particularly sparse and current models of slab geometry (*24*, *26*, *27*) provide a low-resolution smoothed surface with little apparent relationship to segmentation in slip indicators.

**Fig. 1.**
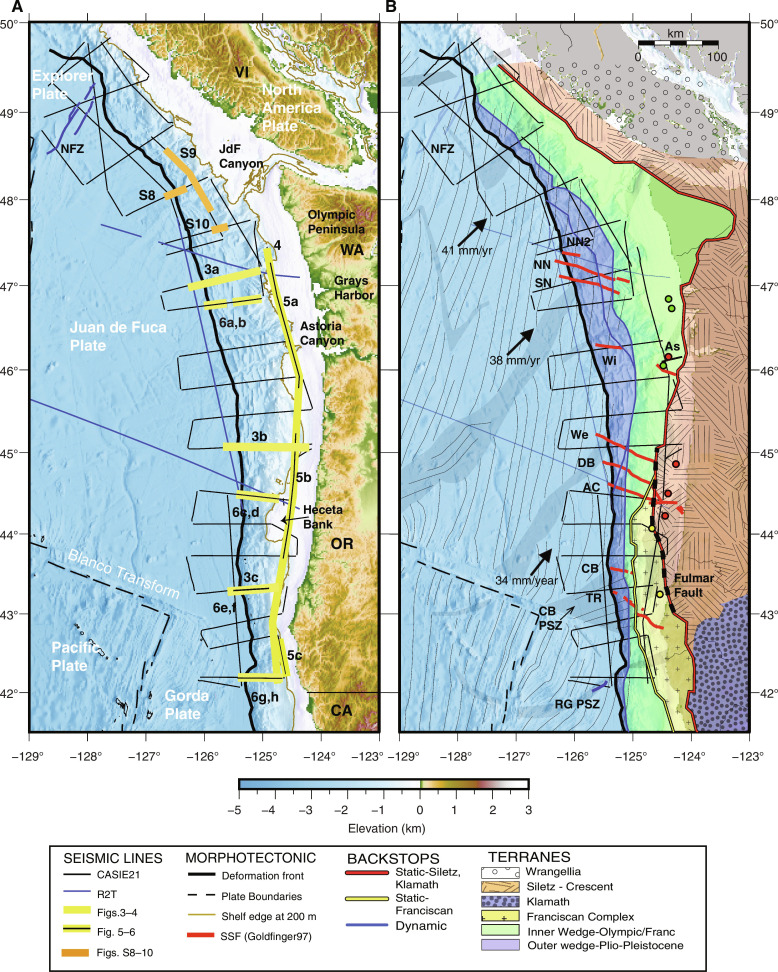
Survey coverage and tectonic framework of the Cascadia Subduction Zone. (**A**) Seafloor bathymetry with seismic tracklines indicated (see fig. S1 for trackline names). Extents of seismic sections shown in Figs. 3 to 6 and figs. S8 to S10 highlighted (see legend) with figure numbers indicated. Tectonic plates labeled and location of primary plate boundaries shown in black dashed line. VI, Vancouver Island; WA, Washington; OR, Oregon; CA, California. (**B**) Tectonic framework including upper plate terranes and backstop locations from (*17*). Fulmar fault (indicated with black dashed line), is interpreted as boundary between Siletz and Franciscan terranes in central-southern Oregon and is modified from (*39*) to follow magnetically defined edge of Siletz from (*17*). Location of industry wells on the shelf from (*39*) shown with colored circles indicating nature of oldest material recovered; red (Eocene basalt), yellow (Lower Eocene arkosic wacke), and green (Upper Oligocene and younger mélange). Lower plate crustal isochrons (thin black lines) and location of propagator shear zones interpreted from offset magnetic isochrons (gray shading) from (*29*); see fig. S1 for isochron ages. Black arrows indicate Juan de Fuca (JdF)–North America relative plate motion (*30*). Primary fault traces of Nootka Fault Zone (NFZ) marking Explorer-JdF (*44*) and reactivated fault within the Rogue Propagator Shear Zone [RG PSZ (*50*)] in blue. Strike-slip faults from (*34*) in red and labeled as follows: TR, Thompson Ridge fault; CB, Coos Basin fault; AC, Alvin Canyon fault; DB, Daisy Bank fault; We, Wecoma fault; Wi, Willapa Canyon fault; SN, South Nitinat fault; and NN, North Nitinat fault. NN2, North Nitinat 2 fault is identified but unnamed in (*34*); As, Astoria Canyon fault is unnamed fault mapped on the shelf from (*39*). CBPSZ, Cape Blanco Propagator Shear Zone.

In this study, we present constraints from a new multichannel seismic (MCS) investigation of the geometry of the downgoing Juan de Fuca (JdF)–Explorer–Gorda plates and plate interface fault within the Cascadia seismogenic zone, providing insights into slip behavior segmentation, and the evolution of plate geometry as it descends. Modern long-offset MCS techniques provide the best tools available for imaging at seismogenic zone depths, revealing the subsurface architecture in high resolution, and providing constraints on material properties along the megathrust that contribute to frictional state. Our study makes use of ~5500 km of data acquired with a 12- to-15-km-long receiver array on a quasi-regular grid composed of intersecting margin crossing and parallel profiles spanning over 900 km along the plate boundary from the northernmost Gorda plate to the northern limit of subduction offshore Vancouver Island. The MCS data were acquired during the Cascadia Seismic Imaging Experiment 2021 (CASIE21), supplemented with ~750 km of data from the 2012 JdF ridge-to-trench study (Materials and Methods and Fig. 1 and fig. S1). Both datasets have been processed to prestack depth migration (PSDM) using advanced methods for noise and multiple suppression, velocity model building, and migration and provide high-resolution structural and physical property information for the plate interface zone, overlying accretionary wedge and downgoing plates. The dataset, along with others acquired as part of the CASIE21 experiment (*28*), support the development of a new structural framework for the offshore margin and is available to the community for other future studies. The results provide greatly increased resolution of the three-dimensional (3D) geometry of the lower plate and plate interface across much of the critical seismogenic portion of the megathrust reaching within a few tens of kilometers from the coast for much of the margin. In key areas, the results suggest substantial differences in plate depth and shape from commonly used current models (*24*, *27*).

### Geologic setting

The CSZ represents a thermally hot end member in the global subduction system where the young incoming JdF-Explorer-Gorda plate system subducts beneath North America. Plate age at the deformation front (DF) is only 4 to 9 million years (Ma) (Fig. 1) (*29*). A series of northeast trending propagator shear zones (PSZs) inherited from crustal formation define the primary segmentation of the lower plate within the subduction zone. The subduction rate is variable, increasing to the north from 30 to 42 mm/year relative to North America and is highly oblique to the margin within the Oregon to Washington regions (*30*). Some of this obliquity is likely accommodated by clockwise rotation of the Southwest Washington and Oregon Coast Ranges, which has been ongoing for the past 50 Ma (*31*, *32*). In the offshore, left-lateral motion along a series of WNW-oriented strike slip faults that offset both plates may also contribute (Fig. 1B) (*33*, *34*). These unusual faults are interpreted as originating in the lower plate due to dextral shearing driven by oblique subduction. Five of these strike-slip faults (SSFs) show clear offsets near the DF in igneous crust and overlying wedge sediments that align with seafloor traces that extend to the upper slope or shelf. These faults form two groups of closely spaced subparallel structures: the North and South Nitinat SSFs offshore Washington and the Alvin Canyon-Daisy Bank-Wecoma SSFs offshore Oregon (*34*). From prior seismic data constraints, the depth extent of other SSFs identified crossing the margin and whether any of these faults rupture the lower plate deeper in the CSZ has been unknown.

The upper plate forearc crust is highly heterogeneous at Cascadia due to the complex tectonic history of this margin over the past 50 Ma [e.g., (*35*–*39*)] and variations in the lithology and structure of the upper plate inherited from this complex history are believed to play an important role in numerous CSZ properties**.** Paleocene to early Eocene age oceanic igneous rocks and volcaniclastics of the accreted Siletz-Crescent terrane are present from Vancouver Island to southern Oregon (Fig. 1B). The western boundary of this terrane extends offshore or along the coast for most of the margin except from 47° to 48.3°N where the western edge of Siletz swings sharply inland due to uplift and erosion within the Olympic peninsula during the Miocene (*31*, *35*–*36*, *40*). Pre-Tertiary rocks of the Klamath terrane are present in the forearc crust south of ~43°N.

Beneath the Oregon continental shelf, wedge sediments and oceanic crust of the Franciscan Complex [pre-Tertiary to early Eocene age; (*17*, *37*–*38*)] are interpreted to be in fault contact with Siletz along the Fulmar fault, a right-lateral SSF active during the Eocene, which may have transported Franciscan Complex rocks north as far as 44.5°N (*38*–*39*). These Eocene to pre-Tertiary age terranes provide primary material strength contrasts forming static backstops along the margin and have been linked to the prominent regional variations in structural style of the accretionary wedge (*17*), as well as patterns of current locking along the plate interface and ETS further downdip (*18*, *21*, *41*–*43*). Secondary, dynamic backstops have also been identified and attributed to the transition between the outer wedge of Plio-Pleistocene sediments and inner wedge of more lithified Miocene and older material. These transitions in wedge sediments represent additional strength heterogeneities, which influence the distribution of active thrust faulting and structural style of the modern wedge.

## RESULTS

### Geometry of the subducting JdF plate and linkages with upper plate structure

**Fig. 2.**
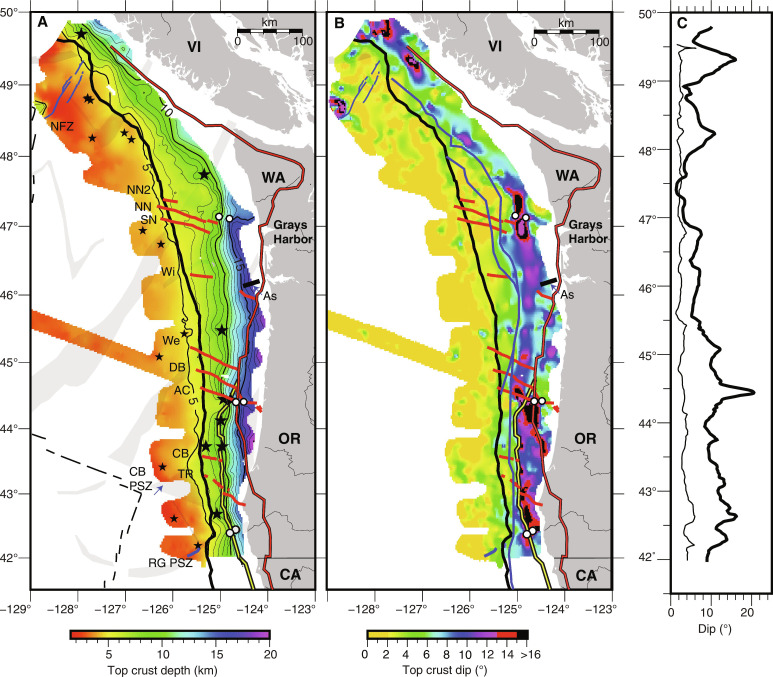
Geometry of top of igneous crust surface along the Cascadia Subduction Zone. (**A**) Gridded surface showing Top Oceanic Crust (TOC) depth derived from CASIE21 and Ridge2Trench Pre-Stack Depth Migration (PSDM) seismic sections. Plate depth contours in thin black line with every 5 km in bold. Apex of seamounts identified from seismic images indicated with black stars. Three pairs of white circles indicate crossing of interpreted faults in lower plate discussed in text. Propagator shear zones (PSZs; gray shading) and plate boundaries (black dashed line) from Fig. 1 are shown to highlight incoming plate tectonic segmentation. (**B**) Calculated dip of TOC from (A). Interpreted western edge of wedge backstops from (*17*) shown including dynamic backstops (blue); static backstops including Siletz-Crescent-Klamath terranes (red with black outline); Franciscan Complex (yellow with black outline). See Materials and Methods for details on gridding and dip calculations. (**C**) Isodistance profiles of dip of TOC at 20 km seaward and 50 km landward of the deformation front (DF) (thin and bold black lines, respectively). Other annotations as in Fig. 1.

**Fig. 3.**
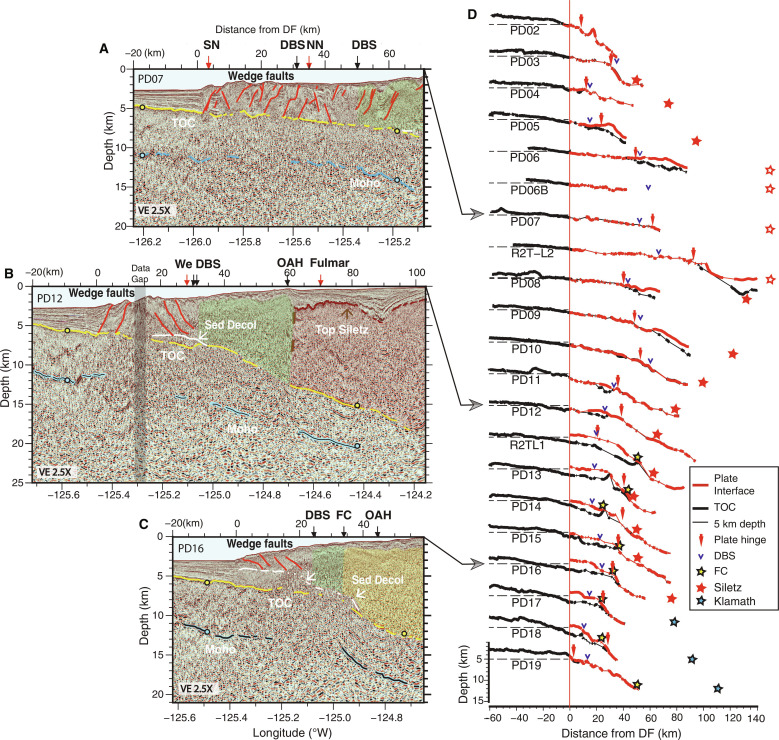
Margin crossing seismic lines illustrating lower plate and plate interface fault geometry. (A to C) Seismic sections with Top Oceanic Crust (TOC), Moho, sediment décollement (Sed Decol) horizons and primary wedge faults identified; sections without interpretations in fig. S4. Horizon depths at line crossings in color-coded circles. Arrows along image top show crossing locations of major faults (red) and morphotectonic features (black) from (*17*); Franciscan Complex (FC), Outer Arc High (OAH), and Dynamic Backstop (DBS); fault names from Fig. 1**.** Location of upper plate terranes defined in (*17*) indicated with colored overlays: Inner Wedge, green; Siletz, red; and Franciscan Complex, yellow (transitions uncertain). See Fig. 1A for profile locations. (**A**) Transect PD07 showing shallowly dipping plate offshore Washington. (**B**) Transect PD12 showing more steeply dipping lower plate with Siletz block in upper plate offshore central Oregon. Top of Siletz terrane interpreted from high-amplitude reflection beneath shelf basin sediments; western edge of Siletz (brown dashed line) interpreted from break and change in character of upper plate reflectivity. Note more westerly location of edge of Siletz than the interpreted Fulmar fault location (*39*). (**C**) Transect PD16 showing steepening in TOC and décollement step down beneath the western edge of Franciscan Complex offshore southern Oregon. (**D**) Stacked profiles of TOC and plate interface horizons from each primary margin-crossing seismic line (Fig. 1 and fig. S1). TOC in black and plate interface in red with horizon picks in bold line and interpolated depths in thin line. Profiles plotted in distance from deformation front (DF) with red vertical line indicating DF (0 km). Vertical depth scale for all profiles at bottom; 5 km reference depth for each profile shown with dashed horizontal line seaward of DF. Colored stars mark location of upper plate crystalline backstops as indicated in legend; open stars denote crustal backstop located further inland than horizontal scale.

**Fig. 4.**
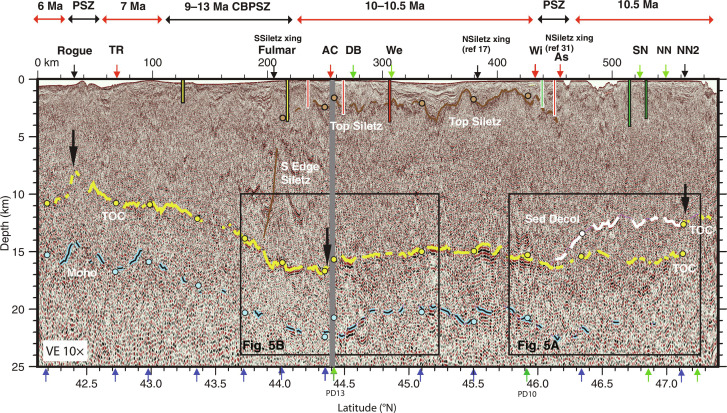
Along-shelf seismic transect PS01with interpreted horizons for Top Oceanic Crust (TOC), Moho, Sediment Décollement and top of Siletz terrane. Seismic section also shown in fig. S5 without interpretations and without automatic gain control applied to highlight more reflective oceanic crust layer, which aids interpretation north of 46°N where TOC and Moho horizons are weak. Vertical colored bars show depth of drill holes along shelf (Fig. 1B and fig. S1) that sample Eocene-age basalt (red), lower Eocene arkosic wacke (yellow), and Upper Oligocene to Miocene mélange (green) from (*39*). Note that drill holes are projected to seismic transect with white/black outline indicating that holes are less than/greater than 5 km from line. Vertical gray bar marks 1.5-km data gap. Approximate age of downgoing plate and location of propagator shear zones (PSZ) from (*29*) at top of image. Horizontal scale at image top is distance from southern end of profile. Vertical arrows along image top/bottom show crossings of previously mapped major faults and seismic dip lines (respectively) (red/blue) or projected to line (green). Faults indicated with black arrows are interpreted in this study. “As” refers to unnamed fault mapped near Astoria Canyon (*39*); other fault name abbreviations as in Fig. 1. Crossings of edge of Siletz terrane (SSiletz and NSiletz xing) shown from both references (*17*) and (*31*). Southern edge of Siletz (brown dashed line) is interpreted from break and change in orientation of upper plate reflectivity and aligns with crossing of Fulmar fault. Large arrows on seismic section indicate steps/disruptions in TOC at fault zones interpreted as important structural discontinuities in regional plate geometry (see text). Horizon depths at line crossings indicated with colored-coded circles [note PD13 and PD10 (labeled) do not cross PS01 but come within 3 km of line and depths at projected line crossings are shown].

**Fig. 5.**
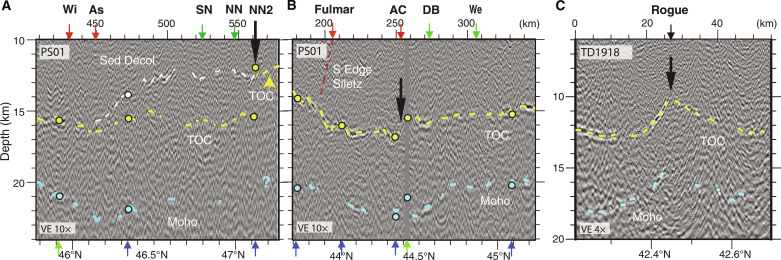
Close-ups of margin-parallel seismic sections under the shelf with interpretations. Seismic section also shown in fig. S6 without interpretations. Other annotations as in Fig. 4. (**A**) Northern section of line PS01 shows offset in crust at location of North Nitinat 2 (NN2) fault, and presence of sediment horizon interpreted as deep slip surface, which extends south from NN2 fault and steps down to Top Oceanic Crust (TOC) near 46°N. (**B**) Central section of line PS01 where Siletz terrane is present in the upper plate and bright TOC and Moho are imaged (see also Fig. 3B and fig. S5). Note the offset in crust at line crossing of Alvin Canyon fault and the step in TOC at southern edge of Siletz block (dashed brown line). (**C**) Line TD1918 from southern Oregon margin showing local shoaling and break in crust at line crossing of the Rogue Propagator Shear Zone (PSZ) fault. Figure 1 shows locations of all panels. In addition, (A) and (B) are shown in Fig. 4.

**Fig. 6.**
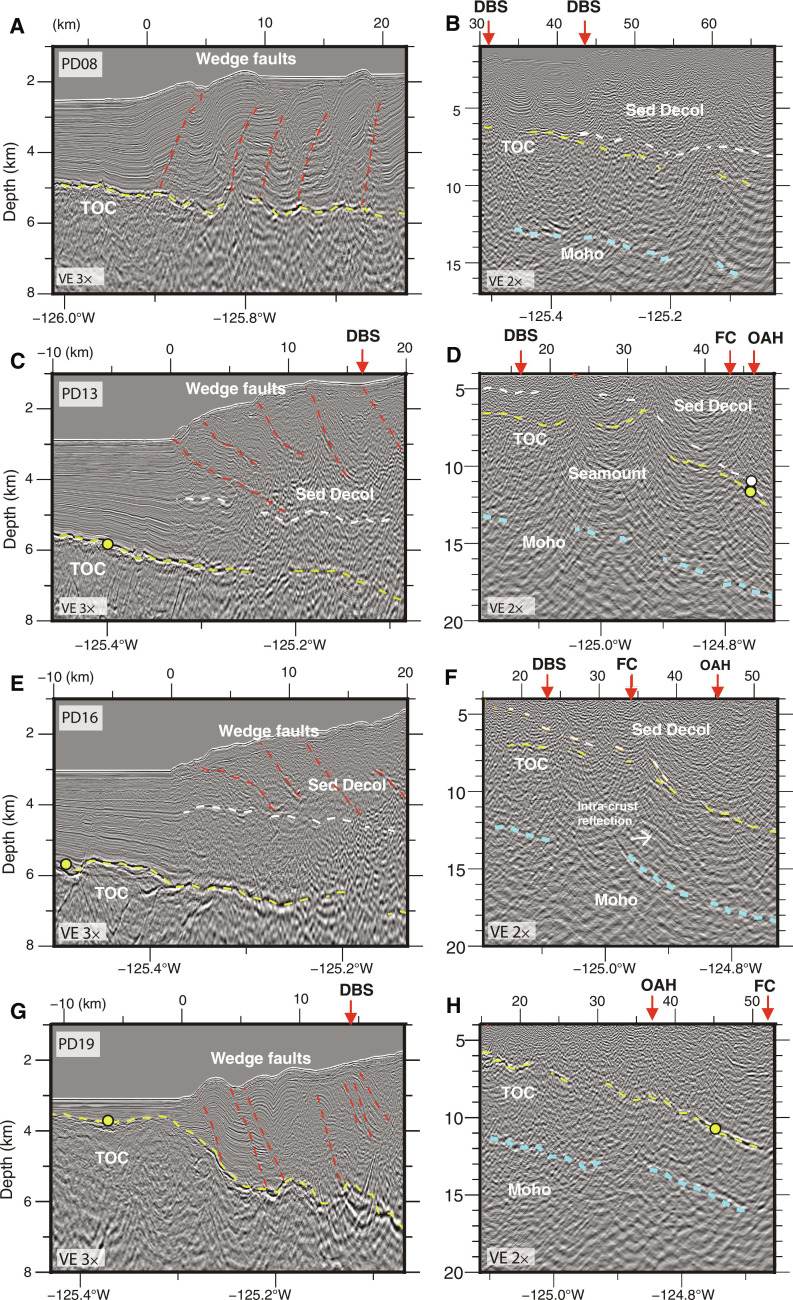
Seismic images from margin crossing transects with interpretations. Left-hand panels span the near deformation front (DF) region along each line and right-hand panels are from the deeper mid slope highlighting Top Oceanic Crust (TOC), Moho, and interpreted sediment décollement, where present, at depth. Seismic sections also shown in fig. S7 without interpretations; line locations in Fig. 1A and line names in fig. S1. Horizontal scale at top of image is distance in km from DF. Arrows along image top show line crossing of interpreted features from (*17*) with annotations as in Fig. 3. (**A**) and (**B**) from seismic transect PD08 offshore central Washington; (**C**) and (**D**) from transect PD13 offshore central Oregon; (**E**) and (**F**) from seismic transect PD16 offshore southern Oregon; (**G**) and (**H**) from transect PD19 offshore southernmost Oregon crossing Gorda plate.

The PSDM seismic images span the CSZ from 42° to 50°N and reveal a seismic reflection of variable amplitude from the top of oceanic crust (TOC) from which a map of TOC depth has been constructed (Figs. 2 to 6, figs. S2 to S10, and Materials and Methods). Regional-scale (hundreds of kilometers) spatial domains with distinct lower plate geometry are evident that align with variations in the distribution of upper plate terranes mapped in prior studies, as well as local heterogeneities in the downgoing plate including seamounts and faults. Below, we describe the characteristics of these domains from north to south and their relationship to overriding plate structures. Our focus is on the large-scale features of lower plate geometry that are well constrained from the acquired seismic grid of margin-parallel and crossing profiles, and fault zones evident from multiple profile crossings that are of particular importance for this regional structure.

Offshore northern Vancouver Island, the Nootka fault zone, which separates Explorer and JdF plates (*44*), coincides with a region of deeper TOC (up to 1 km at the DF) and steeper plate dips (locally, >10°) that extends under the wedge (Fig. 2 and fig. S2). East of Nootka fault zone, TOC depths of ~5 km at the DF are shallower than along most of the margin, consistent with the younger plate age. TOC dip changes near the DF from an average of ~2° on the incoming plate to >4° [Figs. 2C and 3D (PD03-PD05)]. Offshore southern Vancouver Island, within the regional margin bend of the subduction zone, seismic coverage indicates a local trough in TOC depth aligned perpendicular to the margin and an along-margin peak in plate dip under the wedge centered at 48.2°N (Fig. 2, A and C, and fig. S2). A sharp bend in the DF and a marked change in accretionary wedge structure are documented (*17*) at this location, consistent with presence of a structural transition. In the upper plate within this plate segment, the igneous Crescent terrane is present extending offshore underlying the shelf, and the outer wedge is narrow (*35*, *45*).

South of 48.2°N to ~47°N a region of markedly flat TOC is identified with average incoming plate dip angles of 2° continuing under the wedge for over 40 km with no change in plate dip at the DF [Figs. 2 and 3, A and D (PD06-PD08), and fig. S2). This region of flat plate is located offshore the shallow arch in the downgoing plate under the Olympic peninsula inferred in prior models of plate interface geometry and attributed to the concave oceanward bend of the subduction zone from south Vancouver Island to Washington [e.g., (*24*, *46*, *47*)]. In this same region, the upper plate Siletz terrane is located much further from the DF than elsewhere along the margin extending inland up to 100 km east of the coast. Coincident with the landward step in the edge of Siletz at ~47°N, a fault in TOC is inferred on two margin-parallel lines that offsets TOC by 1.8 and 3 km (up to north) under the slope and shelf, respectively (Figs. 4 and 5 and fig. S5). The trend defined by the two fault crossings is in line with a fault strand north of and parallel to the North Nitinat SSF identified in (*34*) that offsets the youngest fold and thrust ridges of the wedge in the same sense. We interpret the lower plate fault as the landward extension of this SSF and refer to it as the North Nitinat 2 fault.

Another change in plate geometry occurs south of ~47°N to ~44.5°N, roughly bounded by the Nitinat SSF system in the north and the Alvin Canyon SSF in the south [Figs. 2 and 3, B and D (PD09-PD12)]. Seismic transects across this region show plate dips of ~4° near the DF. Further landward, a hinge-like narrow zone (up to ~10 km wide) of plate steepening (to >8°) under the wedge is seen at variable distances from the DF defining an arcuate-shaped bending plate in map view (Fig. 2, B and C). The locus of maximum plate curvature zone swings progressively seaward, from ~50 km east of the DF south of the Nitinat faults to only ~20 km near the Alvin Canyon SSF. Further, the location of this local bend in the lower plate tracks closely with the dynamic backstop mapped from seafloor morphology and attributed to the transition from modern outer wedge to more lithified older inner wedge material (Figs. 2B and 3D) (*17*). A marked change in plate geometry across the Alvin Canyon–Daisy Bank–Wecoma fault system coincides with local complexity in the interpreted dynamic backstop configuration consistent with left-lateral motion juxtaposing older wedge material to the north against younger wedge across these SSFs. Offset seafloor structures and sediment horizons indicate that cumulative left-lateral motion of ~10 km across these faults has occurred in the past ~0.65 Ma (*34*). This sense of motion translates older wedge sediments on the north side of the Alvin Canyon–Daisy Bank–Wecoma triplet seaward.

The Alvin Canyon SSF at ~44.5°N appears to be a major discontinuity along the margin with a marked change in plate geometry across this fault (Figs. 2, 4, and 5). While disruption of the lower plate near the DF was reported in prior studies (*34*), the seismic imaging shows evidence for an ~1.5 km shift in average depth of the crust at the projection of this fault to under the shelf, with shallower crust to the north, suggesting this fault zone is a crustal tear across the margin (Figs. 4 and 5B). Bounding the fault to the south are possible buried seamounts. The seamounts are ~1 to 2.5 km high and 5 to 8 km wide (centered at 44.45°N, 124.93°W and 44.44°N, 124.77°W) and are interpreted from weak TOC reflections on two nearby lines [Figs. 2, 3D (R2TL1 and PD13), and 6D]. One of these TOC topographic highs is ~15 km west of a previously identified potential field anomaly attributed to a buried seamount under the wedge (*48*), suggesting that a cluster of seamounts is present in this region. South of these topographic anomalies and extending over 200 km, the trajectory of the lower plate steepens to >12° at near uniform distances of only 25 to 30 km from the DF, coincident with the western edge of the Franciscan terrane [Figs. 2 and 3, C and D (R2TL1-PD18)]. The Franciscan Complex is interpreted to be present west of the Siletz to ~44.5°N, contributing to the static backstop for this portion of the wedge (*17*).

These observations of lower plate steepening coincident with interpreted transitions in upper plate terranes indicate that bending of the young, warm, and hence buoyant JdF plate is highly sensitive to upper plate structure. We attribute the more steeply dipping JdF crust that characterizes the plate south of Alvin Canyon SSF, to greater loading and deflection under the weight of the dense, more rigid Franciscan and Siletz terranes in the upper plate, which extend closer to the DF here than elsewhere along the margin. Similar to observations at the Nankai subduction zone (*13*), both the onset of lower plate steepening and the along strike width of the zone of steeper deeper plate are spatially correlated with the distribution of stronger and denser bodies in the upper plate. North of the Alvin Canyon fault system, local steepening of the lower plate coincident with the dynamic backstop indicates that lower plate geometry is also sensitive to the presumably more modest changes in gravitational loading associated with the transition to more lithified inner wedge rocks (Figs. 2 and 3).

Deeper in the subduction zone, further evidence for close coupling of upper and lower plate structure is found in TOC depth variations beneath the shelf. Along the 590-km margin parallel shelf transect, deepest TOC (15 to 16.5 km) is imaged where the seaward edge of Siletz terrane based on magnetic anomalies and industry drill holes extends offshore or near the coast (from 47° to 43.3°N, Figs. 2 and 4) (*31*, *39*, *48*). This region of deepest lower plate does not simply track with crustal age as shallower depths are found where older crust is present within the Cape Blanco PSZ. A local stepdown in TOC is imaged where our seismic transect crosses the Fulmar fault bounding the Siletz block (Figs. 4 and 5B), and in map view, the southern limit of deepest plate closely follows the landward swing of Siletz (Fig. 2). North of 47°N, the regional arching of the plate under the Olympics due to the concave bend in the CSZ is likely the dominant factor contributing to the markedly flat plate and shallower TOC under the shelf [e.g., (*46*, *49*)]. However, reduced upper plate loads with the far landward location of Siletz are expected and feedbacks are likely given the history of uplift and erosion of Siletz.

At the southern end of our survey, TOC under the shelf shoals to depths <11 km where younger age Gorda plate crust is subducting (Figs. 2 and 4). An abrupt eastward step in the DF is present at 42.3°N coincident with the PSZ in the lower plate that projects onshore to the Rogue River. Seaward of the DF, this shear zone is interpreted from seafloor bathymetry and modern seismicity to be reactivated as a left-oblique fault associated with modern deformation of the Gorda plate (*50*). Beneath the shelf and in line with this fault, both margin-parallel lines crossing this region show a break in the crust centered at the apex of a local shoaling (by 2 km) of TOC and Moho reflections (Figs. 4 and 5C). We attribute this fault and local upward bend of the crust to ongoing deformation of the lower plate under the wedge associated with this reactivated PSZ and refer to this lower plate fault as the Rogue PSZ fault.

### Geometry and properties of the plate interface fault

Because of sparse structural imaging and the lack of seismicity from the offshore CSZ for much of the margin, prior regional-scale models for Cascadia have not been able to distinguish between top of igneous crust and the plate interface fault for the seismogenic portion of the margin (*24*, *27*). The CASIE21 dataset allows for identification of the plate boundary fault from the distribution of wedge structures indicative of compressional deformation, including the geometry and depth extent of thrust faults and presence of folded sediment horizons.

**Fig. 7.**
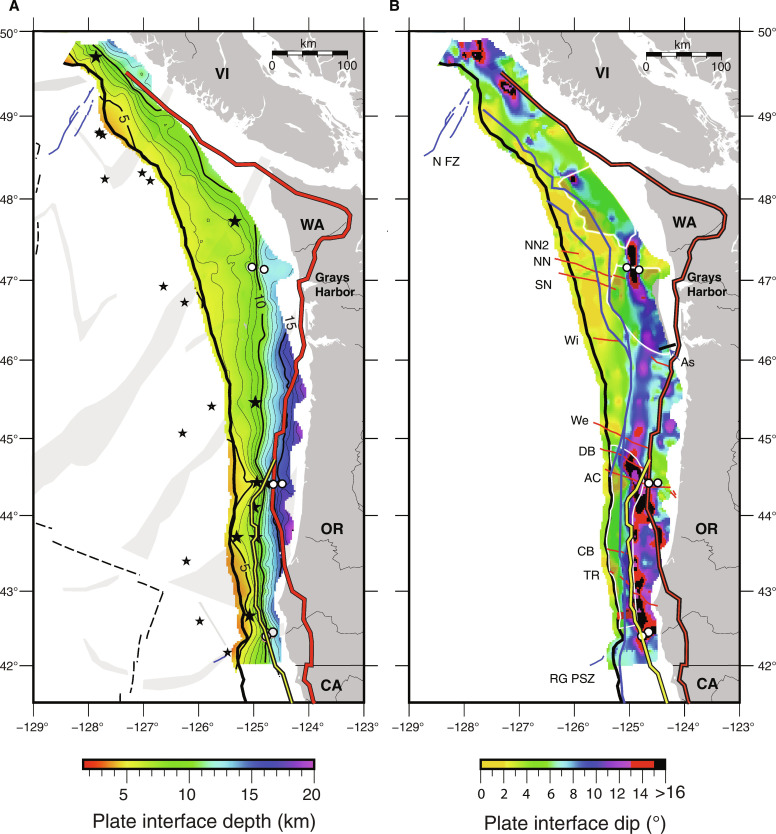
Geometry of plate interface surface along the CSZ. (**A**) Gridded surface showing interpreted plate interface depth from CASIE21 and Ridge2Trench seismic sections. Propagator Shear Zones (PSZs, gray shading) from Fig. 1 shown to highlight incoming plate tectonic segmentation. (**B**) Calculated dip of plate interface surface from (A). White line with semitransparent gray fill shows approximate extent of interpreted décollements within sediment column and highlight where plate interface deviates from top crust surface. Dynamic backstops (blue); static backstops including Siletz-Crescent-Klamath terranes (red with black outline); Franciscan Complex (yellow with black outline) from (*17*). Other features and annotations as in Figs. 1 and 2. See Materials and Methods for details on gridding and dip calculations.

From the northern end of the CSZ to ~44.75°N, seismic images show that frontal thrusts of the modern wedge extend to the TOC reflection (Figs. 3, A and B, and 6A; and fig. S8), indicating that the incoming sediment section is near fully accreted, consistent with observations from earlier studies (*35*, *45*, *51*–*53*). Offshore northern Vancouver Island, wedge faults are well resolved reaching to TOC to 10 to 20 km from the DF. However, offshore southern Vancouver Island, within and extending south of the regional bend in the subduction zone, evidence is found for a sediment décollement within the deeper sediment section indicating a change from full frontal accretion to sediment subduction further downdip. Here, deeper wedge thrusts shoal to a décollement within the sediment section and the TOC horizon below is a markedly brighter and wider horizon consistent with the presence of a package of slower compressional-wave speed, presumably more fluid-rich sediment above (figs. S8 to S10). This deep sediment décollement is interpreted to extend southeast from the sharp bend in the DF at 48.2°N where a possible fault-bounded step in the lower plate topography is imaged under the wedge (Fig. 7 and fig. S9).

Evidence for a change from sediment accretion in the outer wedge to sediment subduction further down dip is found for most of the Washington margin [Figs. 3D (PD06-PD09) and 6, A and B; and fig. S10]. Here, wedge thrusts are imaged reaching to near TOC over a wide region from 30 to 45 km landward of the DF, indicating that the megathrust lies along TOC. However, under the inner wedge and shelf, a subhorizontal reflection above TOC can be mapped across multiple seismic lines that we interpret as a décollement surface within the deep sediments. Near Grays Harbor, a horizon that projects south from the top of the lower plate fault is identified on margin-parallel lines that deepens and merges with TOC near Astoria Canyon (Figs. 4 and 5A). This horizon ties to a reflection on seismic dip lines where it merges with the TOC surface at ~45 to 65 km from the DF (Figs. 6B and 7B). We interpret this horizon as a deep slip surface that formed in response to the North Nitinat 2 fault tear of the JdF plate as a lower angle, perhaps energetically more favorable, slip plane than following the abrupt fault step down in the lower plate. Deep sediment subduction in the Washington region has been inferred from prior seismic imaging (*54*) and is consistent with interpretations of subducted sediments deeper in the SZ beneath the Olympic peninsula (*55*), with a long history of underplating interpreted from the uplift and exhumation of Cascadia accretionary wedge sediments in the Olympic Mountains [e.g., (*31*, *40*)].

In previous seismic studies of the central Oregon margin, a shallow décollement within the sediment column, ~1.5 km above oceanic basement, was identified near the DF south of the Daisy Bank SSF but with unknown downdip and along margin extent due to limited data coverage (*52*, *56*). The CASIE21 data show that this region of shallow sediment underthrusting continues south under the modern wedge for ~250 km along strike to ~42.5°N [Figs. 3, C and D (R2TL1-PD18), and 6, C and E]. The seismic images also show that beneath the older wedge, the sediment décollement horizon steepens and steps down to merge with the TOC horizon at ~35 km landward of the DF (Fig. 6, D and F). The décollement stepdown roughly coincides with the western boundary of the Franciscan Complex in the upper plate (*17*), where increased bending of the lower plate is also observed (Figs. 3, C and D, and 7), with both observations consistent with a prominent density and strength transition in the wedge. The TOC horizon changes reflectivity character at this same location and is notably brighter and more continuous where Franciscan and Siletz terranes are mapped in the upper plate (Fig. 6F and fig. S7f). That the décollement steps down to TOC beneath the Franciscan and Siletz blocks implies little sediment is transported to the deeper subduction zone along this portion of the margin. We infer that the bright top of JdF crust imaged beneath the Franciscan and Siletz blocks reflects a thin fluid-rich sediment layer present between basalts of the lower plate and the crystalline material of the old upper plate terranes.

Sediment underthrusting in the outer wedge offshore central Oregon has been previously linked to subducting seamounts inferred under the shelf in this region, which force the décollement to shift to a plane of weakness in the sediment section up-dip of the seamounts (*48*, *52*). The CASIE21 data indicate the presence of other subducting seamounts beneath the upper slope and shelf, which may be part of a chain or volcanic ridge extending further south under Heceta Bank (Figs. 1 and 7). In addition, our data show a sediment horizon that projects over these basement structures to the shallow sediment décollement that reaches the DF [Figs. 3D (R2TL1-PD15) and 6D]. Numerical modeling studies predict strong modulation of tectonic stresses in the wedge due to seamount subduction with reduced compression on the trailing side of seamounts leading to a zone of less consolidated, higher-porosity sediments that may extend updip for large distances (*57*). We speculate that the deep sediment décollement interpreted to extend south of the basement step up at the North Nitinat 2 fault may reflect analogous stress modulating effects of subducting basement topography. With northeasterly directed subduction, a stress shadow would be expected to the southwest of this several-kilometer-high fault offset in the lower plate, which is oriented at high angles to the subduction direction, and could contribute to the presence of the thick packet of sediment subducting in the trailing wake of this basement topography.

At the southern end of our survey, where northern Gorda plate subduction is imaged, wedge faults extend through the full sediment column to the TOC near the DF and for 16 km landward (Fig. 6G), indicating a return to full frontal accretion of the incoming sediments. The along margin change from outer wedge sediment underthrusting to sediment accretion is likely linked to the complex bulge in lower plate topography imaged under the shelf at 42.5°N across the Rogue PSZ (Fig. 5C). Evidence for additional local zones of sediment subduction linked to lower plate topography are found in the CASIE21 data and are associated with seamounts and basement faults imaged elsewhere under the wedge (e.g., Fig. 3, B and D).

## DISCUSSION

### Insights into paleo-rupture segmentation at Cascadia

**Fig. 8.**
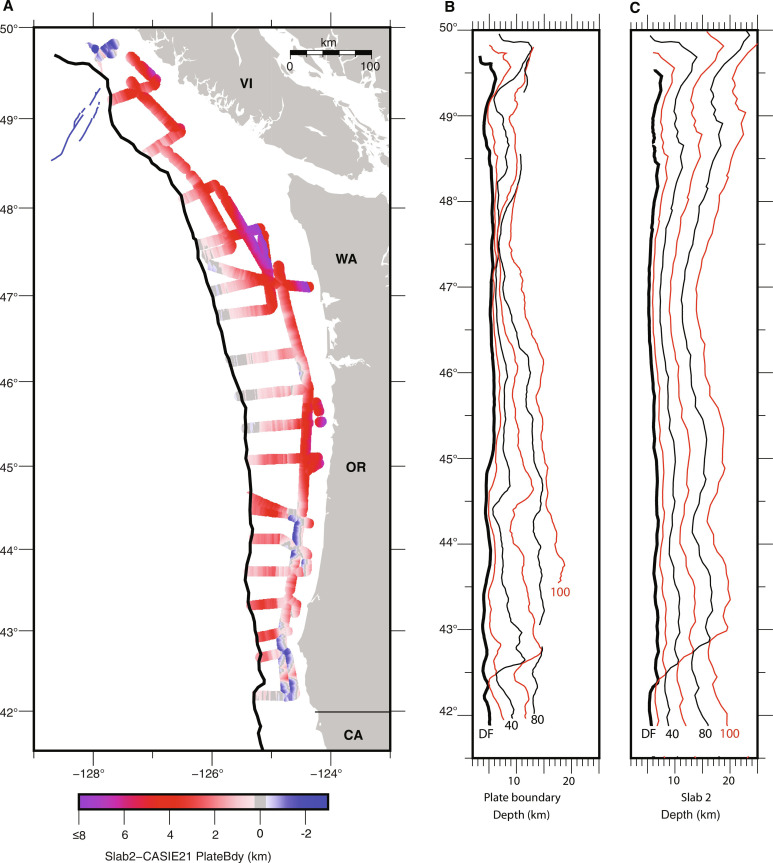
Comparison of CASIE21 plate interface geometry and prior regional plate model. (**A**) Difference between Slab2 depth model (*27*) and plate interface depths from CASIE21 study calculated along survey track lines. Positive values (red to purple) indicate CASIE21 plate model is shallower than Slab2; negative values (blues) indicate our model is deeper. (**B**) Depth to plate interface surface extracted along isodistance profiles spaced every 20 km from DF. Depths along deformation front in thick black line; 40- and 80-km profiles in thin black line with labels; 20-, 60-,100-km profiles in thin red line**.** (**C**) Depth to Slab2 model extracted along same isodistance profiles. Annotations as in B.

**Fig. 9.**
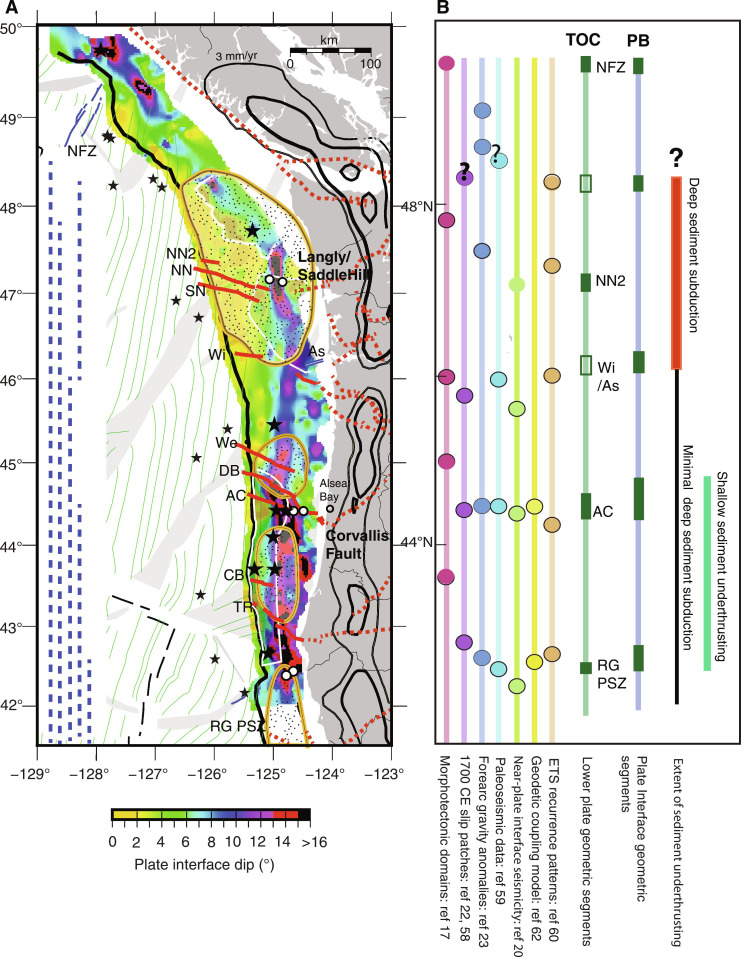
Comparison of geometry of plate interface with paleo-rupture history and slip behavior indicators. (**A**) Dip of plate interface surface from Fig. 7B with modeled extent of high slip (>8 m) patches of the 1700 CE earthquake (*22*) in orange/black polygons with stipple fill, extent of offshore Holocene seismo-turbidites (*59*) in dashed blue lines, and contours of time averaged episodic tremor and slip (ETS) slip rates on land (*60*) in black (30, 10, and 3 mm/year). Also shown are major onshore continental faults from (*41*) in red dashed line and offshore strike slip faults from (*34*) in red. Juan de Fuca plate magnetic isochrons (thin green lines) from (*29*). Other annotations as in Figs. 1 and 2. (**B**) Summary of along-margin evidence for rupture boundaries inferred from diverse geophysical datasets and comparison with transitions in plate (dark green) and plate interface fault geometry (dark blue) found in current study. Also shown are north-south extents of zone of shallow sediment underthrusting and deep sediment subduction identified in this study. Source publications for other geophysical indicators are indicated in column labels. We follow color convention of (*3*), but boundaries are adjusted for the different source publications indicated. Question marks indicate interpreted boundary regions with little data constraints for the parameter characterized.

At Cascadia, broad spatial correlations exist between the interpreted distribution of slip in past earthquakes along the margin and geophysical observations of current plate boundary properties, suggesting the presence of long-term spatially stable rupture patches. While the prevailing view is that upper plate structure and distribution of Siletz terrane plays a primary role in segmentation in plate interface fault properties, observations from the lower plate have been largely missing. Prior slab models that included the offshore region made use of diverse data types with heterogeneous coverage (*24*, *26*–*27*) and poorly suited for investigating the relationship between lower plate structure within the seismogenic zone and paleo-slip indicators along the margin. The CASIE21 observations indicate a plate boundary geometry that is markedly more variable both along and across strike than was evident in prior models (Fig. 8 and figs. S11 to S12). The data reveal the presence of structural segments that differ in slab bending and depth, as well as in the nature of sediments along the plate interface. Further, the observations suggest that differences in upper plate loading of the young and weak JdF plate associated with the distribution of Franciscan and Siletz terranes, as well as older more lithified sediments of the inner wedge, are contributing to these regional variations in lower plate geometry. These observations provide a new framework for interpretation of persistent patches of high slip evident in other datasets and for linking observations of upper plate structure and current frictional state along the plate interface to indicators of paleo-rupture segmentation (Fig. 9 and fig. S13).

#### *Vancouver Island to Washington (49*° *to 46*°*N)*

Paleo-slip models for the 1700 CE earthquake constructed from records of coastal subsidence include a large slip patch extending from offshore Vancouver Island, spanning the full Washington margin, to ~45.5°N (*22*, *58*). Holocene turbidite records indicate ruptures extending to ~48.5°N and that this part of the margin has ruptured less frequently than further south (*59*). However, coastal subsidence and turbidite records are particularly sparse in the north and the size and northern limit of interpreted paleo-slip patches is poorly constrained. Two forearc basins interpreted to reflect possible asperities on the plate interface and the northern half of a third basin is inferred from gravity data [basins A-B and C from (*23*); Fig. 9B] and distinct patterns of ETS characterized by high slip rates are found downdip of this region (*60*).

The CASIE21 data show that the plate interface fault extends as a low dip shallow surface under the slope to near the shelf from 48.2° to 46°N roughly coincident with the high slip (>8 m) portion of the modelled 1700 CE slip patch and compatible with the presence of a rupture patch with large area within this region (Fig. 9 and fig. S13). The plate interface is shallower and flatter in this region than inferred in prior regional models of plate geometry (*24*, *27*), with the largest differences found offshore the Olympic peninsula of over 8 km shallower (Fig. 7 and figs. S11 and 12). Further onshore, receiver function studies (*47*, *61*) indicate plate interface depths under the Olympic peninsula that are up to 10 km shallower than the prior regional slab models, consistent with our findings. Modeling of onshore geodetic data indicates strong seismic locking at present within this portion of the margin (*21*, *62*) and recent observations of plate interface microseismicity suggest that the limit of the locked zone extends further landward in this region than some prior estimates (*20*) (fig. S13), consistent with the shallow flat plate interface imaged in our study.

Global comparisons of subduction zone parameters for large magnitude earthquakes indicate good correlations between flat, low curvature plates and giant earthquakes (*5*, *7*). Both the wider seismogenic zone and more homogeneous shear strength expected along flat megathrusts may promote rupture propagation over large areal extents giving rise to the largest earthquakes. An important finding of our study is that a horizon interpreted as the plate interface transects the deep sediments of the inner wedge offshore of the Olympic peninsula (Fig. 7). This décollement beneath the inner wedge extends downdip of the region of flattest TOC and has latitudinal extents that are aligned with the modeled high slip patch for the 1700 CE event (Fig. 9). Incoming JdF plate basement relief is low and negligible faulting related to bending of the plate is detected seaward of the DF in this region (*63*). With the presence of the deeper slip surface in the sediment column, a smooth plate interface is inferred, which may also contribute to giant earthquake potential [e.g., (*64*, *65*)]. The longer recurrence intervals estimated for this portion of the margin from offshore turbidite records (*19*, *59*) are also consistent with a larger amount of slip per event, related to larger rupture area (*66*). From these observations of plate interface geometry and properties, we infer that the south Vancouver Island through Washington region has greater potential than other sections of the margin for the largest earthquake ruptures. Recent models of tsunami propagation for different earthquake scenarios for the 1700 CE event indicate that partial margin ruptures can fit the tsunami evidence in Japan, although partial rupture centroids located south of 46°N are favored (*67*). Revisiting these scenarios with the more accurate fault geometry constrained by the CASIE21 seismic imaging will be of interest to support future earthquake and tsunami hazard research at Cascadia.

#### *Oregon margin (46*° *to 42.5*°*N)*

**Fig. 10.**
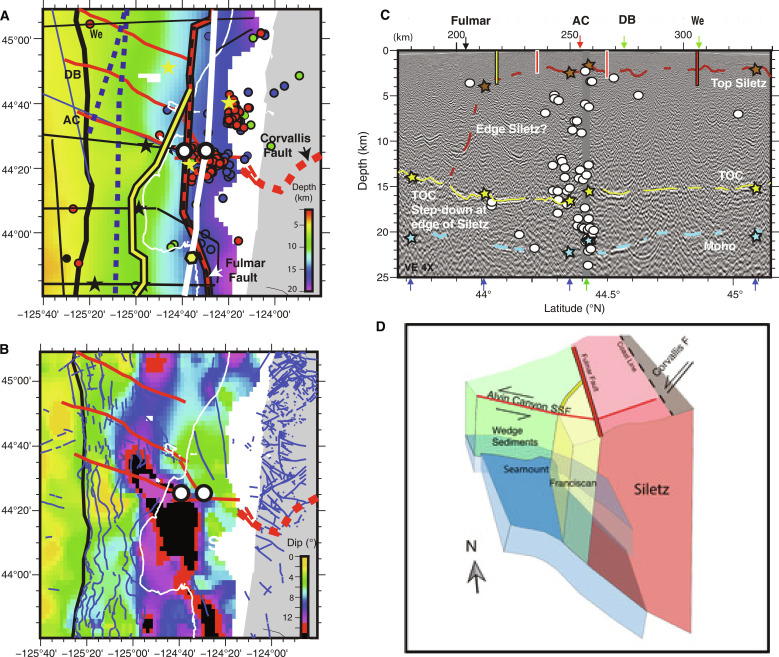
Close-up of Alvin Canyon Strike Slip Fault region. (**A**) Close**-**up map of Top Oceanic Crust (TOC) depth from Figure 2a. Seismic track lines in black with portion of line PS01 in panel C in white. White filled circles with black outline show locations where offsets in downgoing plate are imaged near the intersection of the Alvin Canyon strike slip fault (SSF) with ~east-west oriented fault on shelf. Yellow-filled polygon with black outline shows location of stepdown in TOC coincident with interpreted south edge of Siletz block from panel C. Offshore SSFs from (*34*) in red. Deformation front in medium black line; 200-m contour highlighting the self-edge shown in thin white line. Dynamic backstops from (*17*) in blue dashed line, static backstops in yellow/red with black outline. Onshore Corvallis fault from (*41*, *70*) in thick red dashed line. Seamounts identified from CASIE21 and Ridge2Trench data in black stars. Seamounts identified from potential field data in (*48*) shown with yellow stars. Seismicity catalog from (*20*) is plotted including earthquakes estimated (using Slab2 model) to be from plate interface (red), lower plate (green), upper plate (blue), and incoming plate (black). (**B**) Close**-**up map of dip of TOC surface from Figure 2b. Seafloor and continental faults from (*70*) superimposed in blue. (**C**) Portion of transect PS01 with seismicity from (*20*) located within +/−5 km projected onto profile. Other annotations as in Figure 4. (**D**) Schematic diagram summarizing observations of plate structure in region including left-lateral offset of both plates across the Alvin Canyon SSF, different bending trajectory of Juan de Fuca plate north and south of this fault zone which we attribute to greater gravitational loading to the south with both Franciscan and Siletz terranes present in the upper plate, and relationship with onshore Corvallis fault.

Two high slip patches have been identified offshore Oregon from models of coseismic slip for the 1700 CE event separated by a zone of low slip at ~44.5°N near Alsea Bay where little coastal subsidence is recorded (Fig. 9) (*22*, *58*). Along-strike changes in many other CSZ parameters and local clusters of microseismicity interpreted as a zone of weak coupling attributed to presence of subducting seamounts are also observed at this location (Fig. 10) (*20*, *48*, *68*). The CASIE21 observations indicate a marked change in both TOC and plate interface geometry within this same region coincident with the Alvin Canyon and Daisy Bank SSFs, and we infer that this SSF system marks a major plate heterogeneity along the margin. South of these faults, the region of shallow sediment underthrusting extends for 250 km to 42.5°N, coincident with the southern limit of the south Oregon slip patch. Viscoelastic modeling of onshore geodetic data (*62*) indicates a zone of reduced locking on the megathrust at present from ~44° to 42.5°N, near coincident with the region of shallow sediment underthrusting (Fig. 9). At the northern end of this zone of lower locking, evidence is found for underconsolidation of the sediment package below the shallow décollement with elevated pore fluid pressures and reduced fault strength predicted (*52*). Fluids likely sourced from the plate interface, indicating fluid overpressures, have been sampled in this region (*69*). The seismic imaging indicate that the region of shallow sediment underthrusting extends to the south but is limited to ~30 to 40 km from the DF with a stepdown in the plate interface to TOC at the western edge of old upper plate lithified terranes. This structure creates large downdip gradients in plate interface lithologies, fluid pressures, and geometry, all of which could contribute to the anomalous coupling status inferred in this part of the margin (*21*, *62*). The observations support the presence of a rupture boundary at the Alvin Canyon–Daisy Bank fault system, which is the northern limit of a plate interface segment with distinct geometry and lithology that extends south to ~42.5°N. Prior slab models did not resolve the marked change in plate geometry observed at this SSF system with the CASIE21 data, indicating an up to 2 km shallower plate interface near the DF and more steeply dipping plate further downdip along the southern Oregon margin (Fig. 8 and figs. S11 to S12).

A paleo-rupture segment boundary at ~42.5°N has been inferred in a number of CSZ parameters, including the modeled coseismic slip distribution for the 1700 CE event, the offshore turbidite record, the distribution of forearc basins, and in patterns of ETS onshore (Fig. 9). At this location, the CASIE21 data reveal the presence of a discontinuity in plate geometry centered on a fault in the lower plate under the shelf that is on strike with an active fault documented on the incoming plate within the Rogue PSZ (*50*). The local ~20-km-wide and 2-km-high shoaling of the crust bounding this fault suggests buckling of the plate across the fault zone, compatible with N-S compression inferred from seafloor structures and seismicity in the interior of the Gorda plate/deformation zone. A change in the stratigraphic level of the décollement near the DF is also observed in this region, with shallow sediment underthrusting to the north of 42.5°N and full frontal accretion to the south (Figs. 3, 6, E and G, and 7). These observations indicate an abrupt transition in both plate interface geometry and lithology, consistent with a geologically controlled rupture boundary in this region. The data coverage limits our ability to characterize the rest of the Gorda plate portion of the CSZ [e.g., (*22*, *23*)] and future studies will be needed to characterize plate morphology to inform earthquake hazard studies for northern California.

### Fragmentation of the lower plate under the wedge

A key finding of our study is that the previously mapped WNW SSFs that accommodate some component of oblique convergence along the Washington and Oregon margins are playing an important role in regional plate geometry, with the Nitinat and Alvin Canyon fault systems bounding crustal blocks with distinct geometry. The SSFs, which offset the crustal section of the JdF plate in our images, must be substantial tears within this young, buoyant, and weak plate to facilitate differential bending of adjacent oceanic crustal blocks in response to contrasting local loads and stresses.

The margin oblique SSFs found transecting both upper and lower plates at Cascadia are unusual; at other subduction zones convergence obliquity is commonly accommodated by development of margin parallel SSFs in the upper plate (e.g., the Semangko Fault in Sumatra or Median Tectonic Line in Japan). The Cascadia faults have been interpreted as Riedel-type secondary shears in a dextral shear couple along the margin due to oblique subduction, with the age of faulting estimated at 0.6 Ma (*33*, *34*). The WNW trending SSFs systems cross-cut but do not align with the trend of PSZs in the lower plate, which are the preexisting zones of weakness that define the primary lower plate segmentation within the subduction zone (Fig. 1B). An important question is why these faults nucleate where they do. Several observations from our study indicate the structure of the upper plate forearc may be playing an important role in the nucleation of these faults. The North Nitinat 2 fault identified under the shelf and upper slope near Grays Harbor underlies the projected intersection with the ~E-W trending Langley/Saddle Hill faults mapped on the shelf (*70*). Onshore, these faults connect to a major continental fault system that juxtaposes igneous Siletz terrane to the south and Olympic accretionary complex to the north [e.g., (*31*)]. The sense of fault offset in the lower plate under the shelf, up to the north, is consistent with lower vertical load from the upper plate associated with the presence of less dense Olympic accretionary complex rocks to the north. Observations of the location of oceanic Moho further onshore derived from receiver functions indicate a step up to the north coincident with the northern boundary of Siletz, indicating a lower plate tear coincident with the edge of Siletz terrane is also present deep within the CSZ (*61*).

Similar relationships between upper plate structures and lower plate deformation are evident at the Alvin Canyon SSF. The landward projection of the Alvin Canyon fault intersects an ~E-W trending fault on the shelf that intersects the Corvallis Fault, another major continental fault onshore (Figs. 9 and 10). Seismic imaging indicates a change in the internal reflectivity of the Siletz block coincident with the E-W shelf fault and offset sediment horizons in the shallowest section, and we infer two blocks of distinct structure are offset along this fault (Fig. 10). A 1.5-km step in the depth of the lower plate crust occurs at this same location, and the sense of offset, deeper to the south, is consistent with greater loading and deflection of the lower plate along the south Oregon margin where both Siletz and Franciscan terranes are present in the upper plate to the mid slope. The abundant microseismicity detected in this region and attributed to the nearby seamount mapped under the slope (*20*, *25*, *48*) projects to our seismic profile along the interpreted fault traces in both upper and lower plates (Fig. 10). Although estimated uncertainties in event depths in this region are considerable (~1 to >3 km) (*20*), this finding is intriguing and suggests that microseismicity in this region may be linked to motion on this fault, possibly in addition to interaction of nearby seamounts with the western edge of Siletz. Directly southeast of this microseismicity cluster and in line with the trend of the Alvin Canyon fault, a narrow high compressional wavespeed (Vp)/high density anomaly is imaged within the Siletz block and interpreted as a dike feeder zone for the late Eocene age Yachats basalts (*14*). A 3D velocity and Moho surface model that overlaps the eastern edge of our survey from 43.8° to 44.8°N shows a local deflection of 2 to 4 km in the lower plate attributed to loading beneath this high-density dike feeder zone. We speculate that the Alvin Canyon fault may also be related to this structural complexity.

The Willapa and Thompson Ridge SSFs project to offshore extensions of major forearc block boundary faults and show similar angular relationships with onshore fault systems (Fig. 9). Although it is unclear from our data whether the Willapa SSF extends east of the lower slope, the trend of this fault projects to the shelf where the ~E-W fault along the Astoria Canyon is identified at ~46.2°N (fault labeled as As; Figs. 1 and 9) (*49*). The interpreted top Siletz horizon terminates at this fault (Fig. 4), and we interpret Eocene Siletz block to south and younger wedge sediments to north across this fault. The deep sediment décollement identified along the Washington margin steps down to TOC roughly coincident with this fault suggestive of a strength barrier in the upper plate associated with a change in upper plate lithology. Evidence for a fault discontinuity in the lower plate is also found at this location including a local deepening of TOC and Moho reflections and a marked change in the coherence and amplitude of both reflections.

The block boundary faults found onshore accommodate northward translation and clockwise rotation of forearc blocks, consistent with the sense of motion across the offshore system, and a wide zone of dextral shear of the North American margin documented by GPS studies (*32*). Strong spatial correlations are found between these forearc faults and zones of low ETS density along the margin. Fluid drainage from the plate interface along these continental faults may dampen tremor forming ETS segment boundaries and possibly contributing to rupture segmentation further up-dip (*41*)**.** Our observations support these prior inferences that the forearc faults and megathrust act as an integrated system but also suggest mechanisms linked to the development of the offshore SSFs that fragment and segment the lower plate**.** These SSFs raise many important questions concerning the structural evolution and deformation of the wedge and lower plate, including how the block rotations required by motion on these subparallel faults are accommodated over time, the nature of strain transitions at the ends of these faults, and the frictional coupling between upper and lower plates (*33*–*34*). Early studies of these fault systems, based primarily on structural observations of the shallow wedge section, provide insights into these questions and indicate relationships with complex strain patterns on the shelf where structures indicative of N-S–oriented compression and local zones of uplift and erosion are found (*71*). Observations indicating that these SSF are likely sites of abundant fluid drainage from plate interface depths (*33*, *69*, *72*), and our findings that these faults are boundaries between crustal segments with distinct geometry and plate interface properties, indicate that they must play an important role in along-margin rupture segmentation at Cascadia, possibly as rupture barriers along the margin. These features are intriguing targets for future investigation.

The picture emerging from the CASIE21 structural imaging is of variable bending and fracturing of the incoming JdF plate system in response to the regional tectonic stresses driving subduction, modulated by the heterogeneous terrane structure of the upper plate. Studies of incoming plate structure within the outer rise at other subduction zones indicate that substantial weakening of the plate occurs because of brittle fracturing and hydration of the plate seaward of the trench as subduction bending begins [e.g., (*11*)]. The lower plate will continue to weaken with ongoing deformation and plastic yielding as it descends beneath the overriding plate giving rise to rheological weakening of the slab at depth (*73*). With negligible plate bending in the Washington region under the modern wedge compared with central Oregon, substantial variability in plate strength along the Cascadia margin is expected further contributing to rupture segmentation. An important insight from our study is that segmentation in both the lithology and geometry of the plate interface, bounded by structural heterogeneities associated with fault zones in the lower plate, can develop within the seismogenic zone, as the lower plate bends and deforms in response to a heterogeneous upper plate. Given its young age, the weak and buoyant JdF plate may be particularly sensitive to upper plate structure as the thin elastic plate bends, and tears develop in the plate under variable upper plate loads. Similar regional-scale characterization of subduction zone architecture from other systems is needed to make further progress on understanding the nature and persistence of rupture segmentation elsewhere and linkages with evolving lower plate morphology as the plate bends in the subduction zone**.**

## MATERIALS AND METHODS

### MCS data and processing

MCS data used in this study were acquired during R/V *Marcus Langseth* expedition MGL2104 with data collected along a quasi-regular grid of 18 primary dip lines crossing the margin (PD02-PD19), seven primary strike lines parallel to the margin (PS01-PS07), and 16 turn lines (fig. S1) (*74*). Survey coverage extended from the northernmost Gorda plate at 42°N to the northern limit of subduction offshore Vancouver Island, spanning ~900 km along the CSZ. Original survey goals included the California portion of the CSZ but were not pursued due to regulatory-related challenges for this part of the margin. Strike lines were shot along the shelf or near shelf edge and 10 to 20 km seaward of the DF to characterize the downdip portion of the seismogenic zone and the incoming plate just prior to subduction. Dip lines were located to sample the primary segmentation evident in different subduction zone characteristics at Cascadia, fill major data gaps in prior data coverage, and support a regional-scale characterization with approximately regular line spacing of 50 to 75 km. The MCS data were acquired using a tuned ~108.2 liters (6600 in^3^) airgun source array with a 37.5-m shot interval, and a 12/15-km-long (960/1200 channels) hydrophone streamer with 2-ms sample interval and 15-s record length. The survey was conducted with marine mammal mitigation procedures in place and environmental permits that defined where data could be collected along the shelf. The 2021 survey coverage is augmented with data acquired during the 2012 JdF Ridge to Trench experiment (cruise MGL1211), which included two margin-crossing transects and a margin parallel strike line seaward of the DF that complements the 2021 coverage. The 2012 data were acquired with a 6600 in^3^ source and 37.5- and 50-m shot intervals, recorded on an 8-km, 636 channel streamer with 2-ms sample interval and 12.3-s record length. Further details on MGL1211 acquisition are previously described in (*63*).

For our study, both datasets were processed by ION Geophysical using the same processing sequence with three primary phases: preprocessing, PSDM, and postprocessing. Preprocessing steps included geometry merge and quality control, geometrical spreading correction, de-bias and de-bubble filter, multiple processes for noise attenuation (swell noise attenuation, seismic interference attenuation, and radial filter for residual noise attenuation), acquisition footprint removal to boost low-amplitude channels and shots, de-ghosting and residual de-bubble, zero-phase conversion, multiple attenuation [including short period multiple attenuation for shallow water data (<1100 ms), free surface–related multiple elimination, radon, and apex-shifted multiple attenuation], and phase-only Q. The data were then resampled to 4 ms, trace decimation with spatial anti-aliasing was applied, lines with multiple segments were merged, with another application of residual noise attenuation applied, followed by time variant band pass filter, and removal of geometric spreading correction.

PSDM processing included velocity model building, residual moveout correction, and anisotropy correction. Velocity model building for PSDM included four iterations of reflection tomography which provides excellent constraints on velocities for the incoming plate sediments and for the accretionary wedge to the maximum depth of coherent reflections. Full waveform inversion (FWI) of refracted arrivals recorded on the streamer was conducted for the deep-water portions of the sections (>1 km) and used to update reflection tomography Vp models. FWI models had the most impact on imaging faults and structure in the shallow 1 to 2 km of the oceanic crust seaward of the DF. For deeper parts of the wedge, velocity constraints from three other sources were incorporated. Vp models derived from streamer tomography of the CASIE21 MCS data (for lines PD09, PD12, PD13, PD16, and PD19; fig. S1) were merged below the reflection tomography models and added constraints for additional ~1 to 3 km. The 3D Vp model of (*14*) derived from travel-time tomography of wide-angle refraction data was used for central Oregon lines from ~44° to 45°N (PD13, PD14, and part of PS01). For all other lines and for deeper sections of lines with streamer tomography models, the USGS 3D Vp model of (*75*) was used. For the oceanic crust, a 1D average Vp model derived from the 2D OBS transect offshore central Oregon (*76*) hung from TOC was used as the starting model with Vp updates for optimal imaging of Moho during a final iteration of the reflection tomography. Vertical transverse isotropy anisotropy was incorporated in velocity model building of sediment section on the incoming plate and accretionary wedge. Thomsen parameter epsilon was derived from scans on final isotropic depth gathers and delta was derived by scaling epsilon by 0.5. Delta is in general less than 5%. Seismic anisotropy effect is the most prominent in the basal sediment layer on the incoming plate.

Postprocessing was conducted on the migrated residual moveout corrected common midpoint gathers and included depth-to-time conversion, high-resolution radon de-multiple, linear noise attenuation, application of 0° to 30° angle mute, stack, inverse Q, time-variant band pass filter, signal coherency enhancement, automatic gain control (AGC)–type amplitude scaling, time-to-depth conversion, and output to SEG-Y format. Further details on data processing including processing parameters and application examples are described in the ION Geophysical Data Processing Report provided with the final PSDM sections archived at the Marine Geoscience Data System (*77*).

### Horizon interpretation and gridding

All PSDM sections were loaded into the IHS Kingdom Suite software package for interpretation. Seismic sections with and without AGC and both depth and depth-to-time sections were used to aid interpretation. The TOC crust horizon was identified on the basis of reflection characteristics including amplitude, wavelength/frequency, and continuity. Moho reflections and other intracrustal reflectivity were used to aid in top crust interpretations. Digitized horizons were also converted to two-way travel time and compared with TOC reflections evident in publicly available legacy shorter streamer MCS data including Canadian Geologic Survey data acquired in 1985/1989 offshore Vancouver Island (*35*, *45*), the 1996 Sonne survey S0108 offshore Washington (*51*, *54*), and the 1989 Geotide survey GT8909 offshore central Oregon (*56*).

The plate boundary interface is identified from the geometry and depth extent of faults transecting the sediment package and the geometry of deep sediment horizons and is well constrained near the DF for the full margin. Where frontal thrusts faults shoal into a décollement within the sediment column, observations of underlying horizons subparallel to TOC indicate they have bypassed wedge deformation and help extend interpretations downdip. Observations of changes in TOC reflectivity beneath interpreted sediment décollements further support interpretations (see text). Sediment underthrusting near the DF along the central Oregon margin was previously identified as terminating at the Daisy Bank strike slip fault with a stepdown to near TOC north of this fault (*56*). We include estimated depth to plate interface based on these earlier data as defining the northern limit of sediment underthrusting in our interpretations.

For digitizing horizons, Kingdom’s guided automated picking tool was used where reflections were sufficiently continuous and bright, with manual picking elsewhere. The center of first positive peak of the reflection waveform was digitized except for the few locations where the horizon was clearly of reversed polarity. Digitized horizons were gridded using tools of the GMT package (*78*). The dense input picks (minimum 12.5 m spaced where horizons are identified) were averaged using blockmedian to avoid aliasing at short wavelengths, the minimum curvature algorithm surface was used to construct a filled smoothly varying grid beneath the wedge (Figs. 2A and 7A), and nearneighbor was used for gridding along track lines (fig. S2). For all grids, a 2.5-km grid interval and 10-km search radius was used; tension factor = 0 was used for the surface grid. Final minimum curvature grids for TOC were masked beyond ~5 km from track lines on seaward and landward sides of data coverage and at the DF for the plate boundary grid. The module grdgradient was used to calculate the magnitude of the vector gradient for TOC and plate boundary shown in Figs. 2B and 7B.

We estimate uncertainties in reflector depth by varying the Vp model by ±5% on one margin crossing transect and rerun Kirchhoff PSDM (fig. S3). These velocities likely overestimate the uncertainties on the incoming plate but still show sensitivity to these velocity changes at depth. From this analysis, we estimate depth uncertainties of ~±50 m on the incoming plate to ~±900 m under the shelf. The increase in uncertainties with increasing depth under the wedge is due to the lack of deep coherent reflectivity to constrain the reflection tomography and the faster Vp at depth and hence less moveout to constrain velocities.

## Supplementary Materials

### This PDF file includes:

Figs. S1 to S13 References
