# Supplementary material for: Subducting plate structure and megathrust morphology from deep seismic imaging linked to earthquake rupture segmentation at Cascadia
Source: Sci Adv. 2024 Jun 7;10(23):eadl3198. doi: 10.1126/sciadv.adl3198 (PMC11160458; doi:10.1126/sciadv.adl3198)
Supplement: Supplementary file 1 — Figs. S1 to S13 References [file sciadv.adl3198_sm.pdf]

Science Advances
AAAS

Supplementary Materials for

Subducting plate structure and megathrust morphology from deep seismic imaging linked to earthquake rupture segmentation at Cascadia

Suzanne M. Carbotte *et al.*

Corresponding author: Suzanne M. Carbotte, carbotte@ldeo.columbia.edu

Sci. Adv. **10**, eadl3198 (2024)
DOI: 10.1126/sciadv.adl3198

This PDF file includes:

Figs. S1 to S13
References

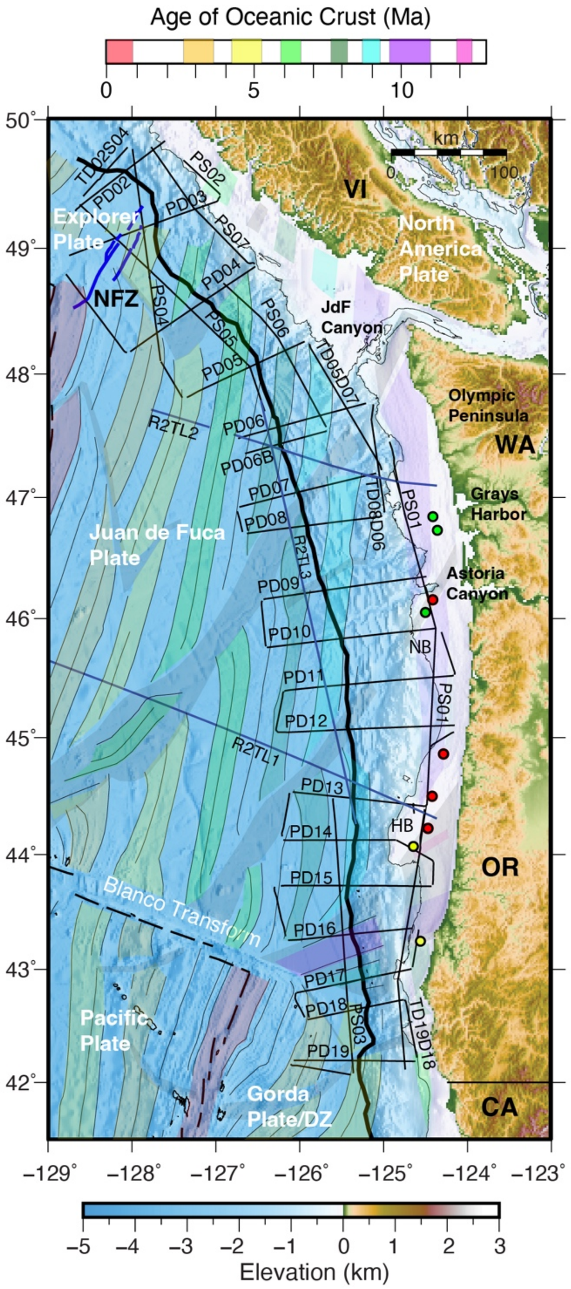

Figure S1. Regional topography with experiment tracklines and line names indicated. Location of industry wells on the shelf (39) shown with colored circles indicating nature of oldest material recovered; red (Eocene basalt), yellow (Lower Eocene arkosic wacke), green (Upper Oligocene and younger mélange). Mid-ocean ridges and Blanco transform fault location shown in light dashed line. Lower plate crustal age and location of propagator shear zones interpreted from offset magnetic isochrons (grey shading) from (29); note that crustal age interpretation and propagator shear zones under the upper slope and shelf are inferred in (29) from magnetic isochrons on conjugate Pacific plate and are shown in fainter colored polygons to indicate lower confidence. Shelf features HB-Heceta Bank; NB- Nehalem Bank. Other annotation as in Figure 1.

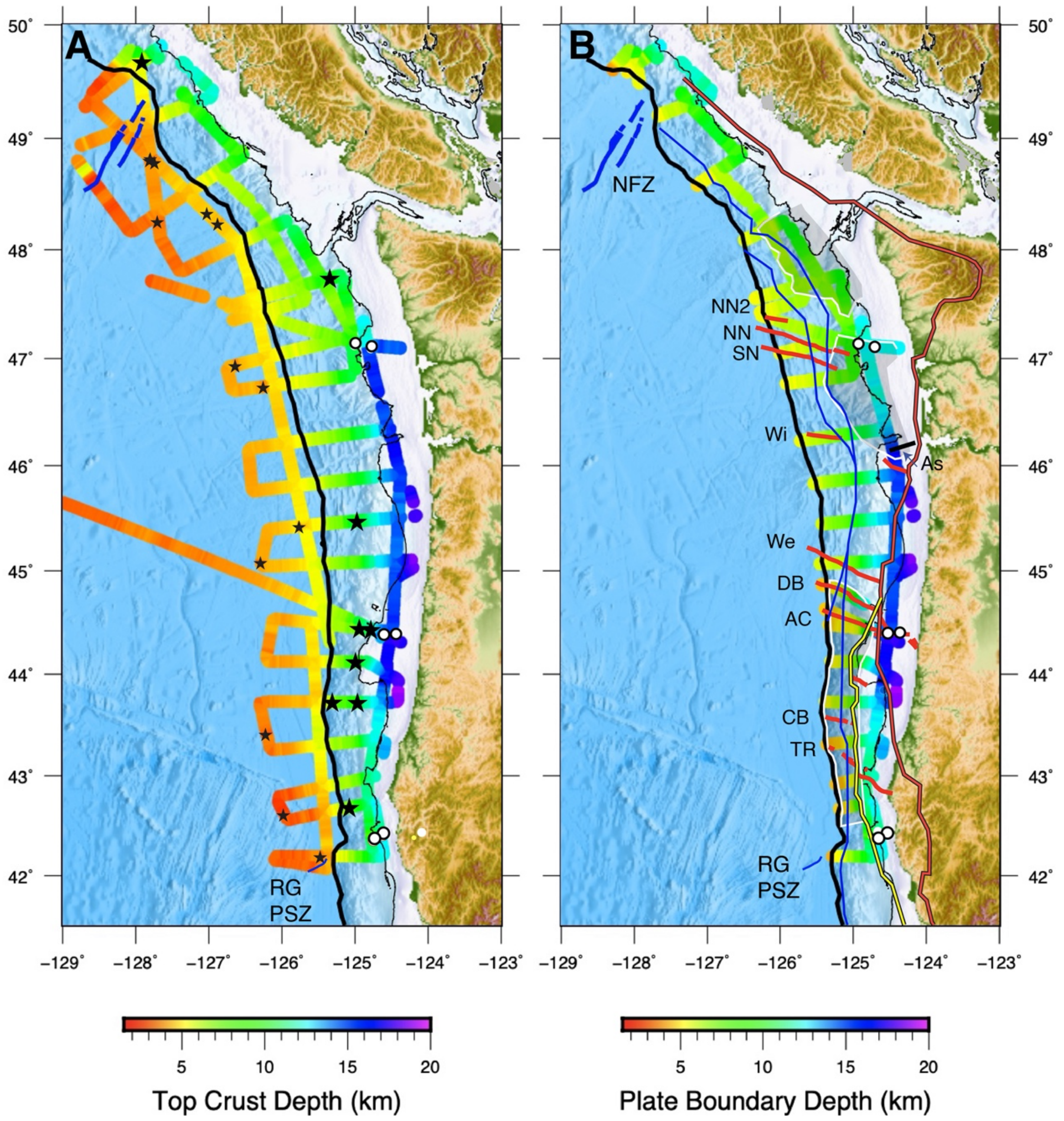

Figure S2. **(A)** Gridded surface showing depth to top of crust derived from CASIE21 and Ridge-to-Trench PSDM seismic sections along tracklines superimposed on regional bathymetry. Apex of buried seamounts identified from seismic data indicated with small (incoming plate) and large (under accretionary wedge) black stars. **(B)** Gridded surface showing depth to interpreted plate interface along tracklines and along Daisy Bank fault derived from (56) as described in Methods. White polygon with grey transparent fill shows approximate outline of interpreted regional-scale décollements within sediment column and highlight primary regions where plate interface deviates from top crust surface. Three pairs of white circles indicate crossing of faults in lower plate discussed in text. Deformation front is shown in bold black line; 200 m contour defining the shelf edge indicated in thin black line. Other annotations including strike-slip faults and upper plate backstops discussed in text are as in Fig. 2.

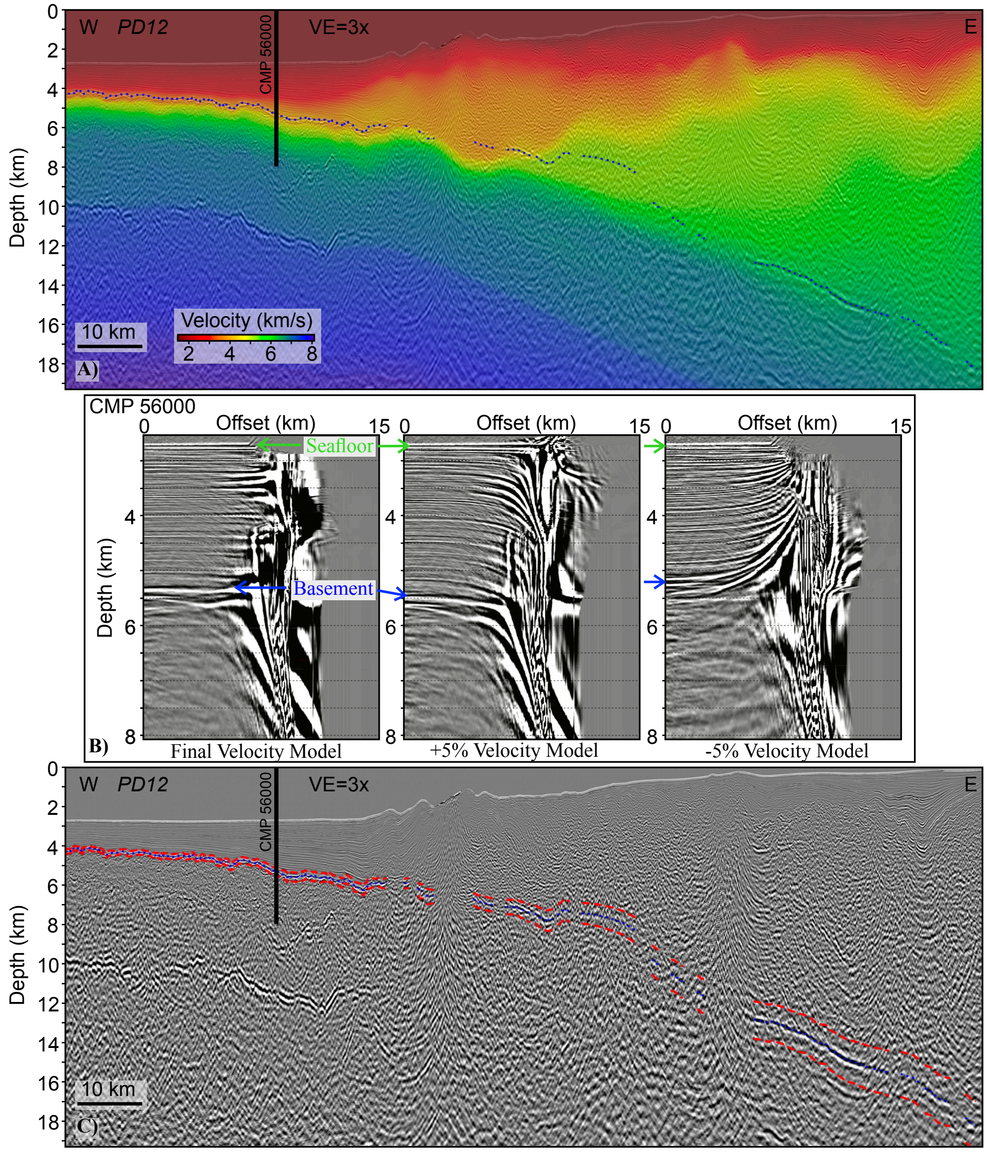

Figure S3. Velocity error analysis from Kirchhoff pre-stack depth migration. **(A)** Final velocity model overlaid on the final depth stack for line PD12. Blue dotted line is the interpreted top of crust; the location common midpoint (CMP) gather in **(B)** in thick black line. **(B)** Results from Kirchhoff pre-stack depth migration showing CMP gather 56000 using three different velocities: the final velocity model shown in A), +5% of the final crustal velocity model, and -5% of the final crustal velocity model. The flatten migrated CMP gathers using the final velocity model flatten the top crust basement horizon while the ±5% velocity models over or under migrate the data, indicating this Vp range overestimates the sensitivity of the velocity model. **(C)** The interpreted top of crust (blue line) and interpretations depth converted using the ±5% velocity models (red lines) give the range of possible depths for the top of crust of ~±50 m on the incoming plate to ~±900 m under the shelf.

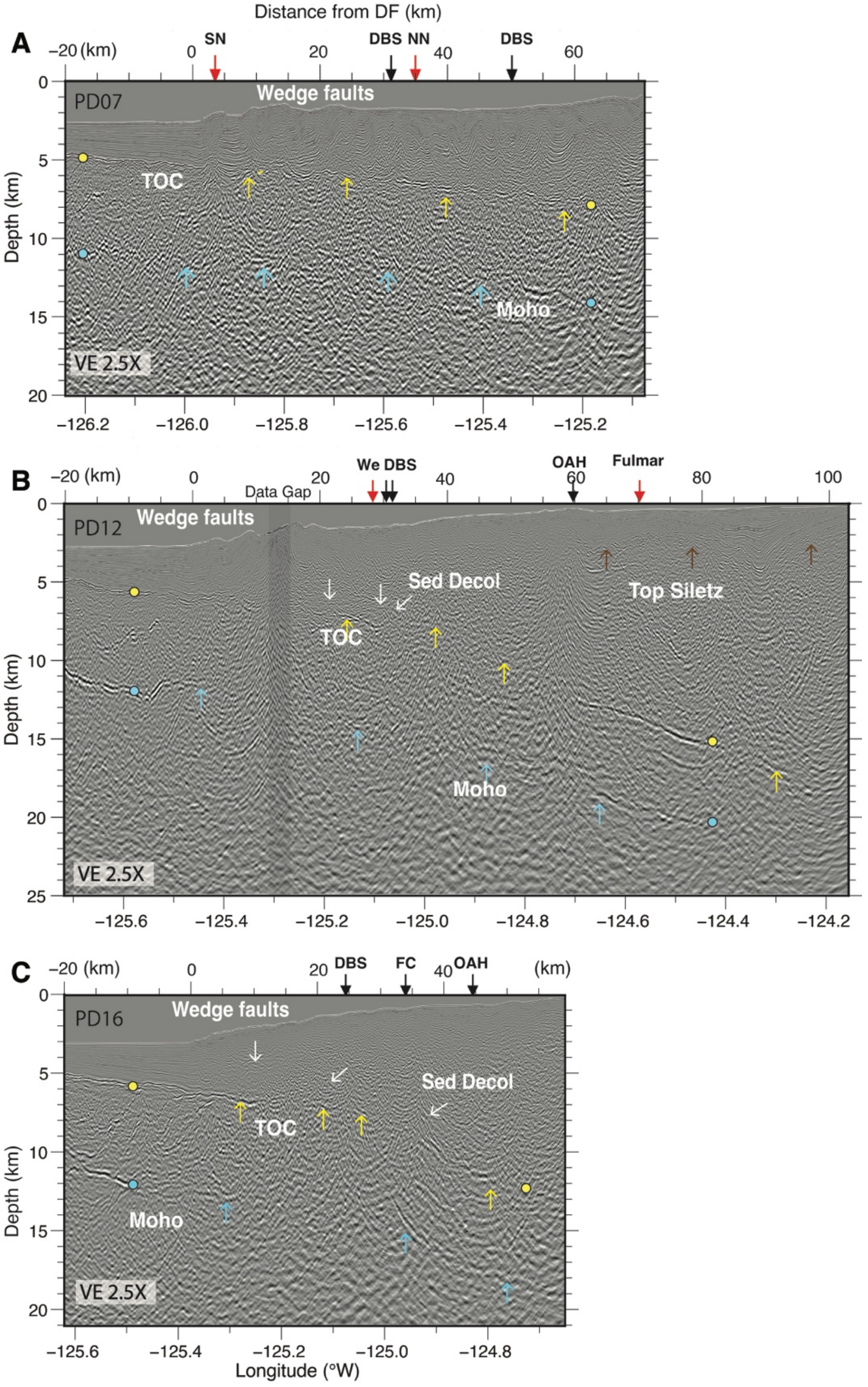

Figure S4. Margin crossing seismic transects from Figure 3 displayed in black-white color scheme without horizon interpretations shown. Horizon depths at line crossings indicated with circles colored coded for each horizon, colored arrows are included to help identify the reflections interpreted from Fig. 3. Other annotations as in Figs. 1 and 3.

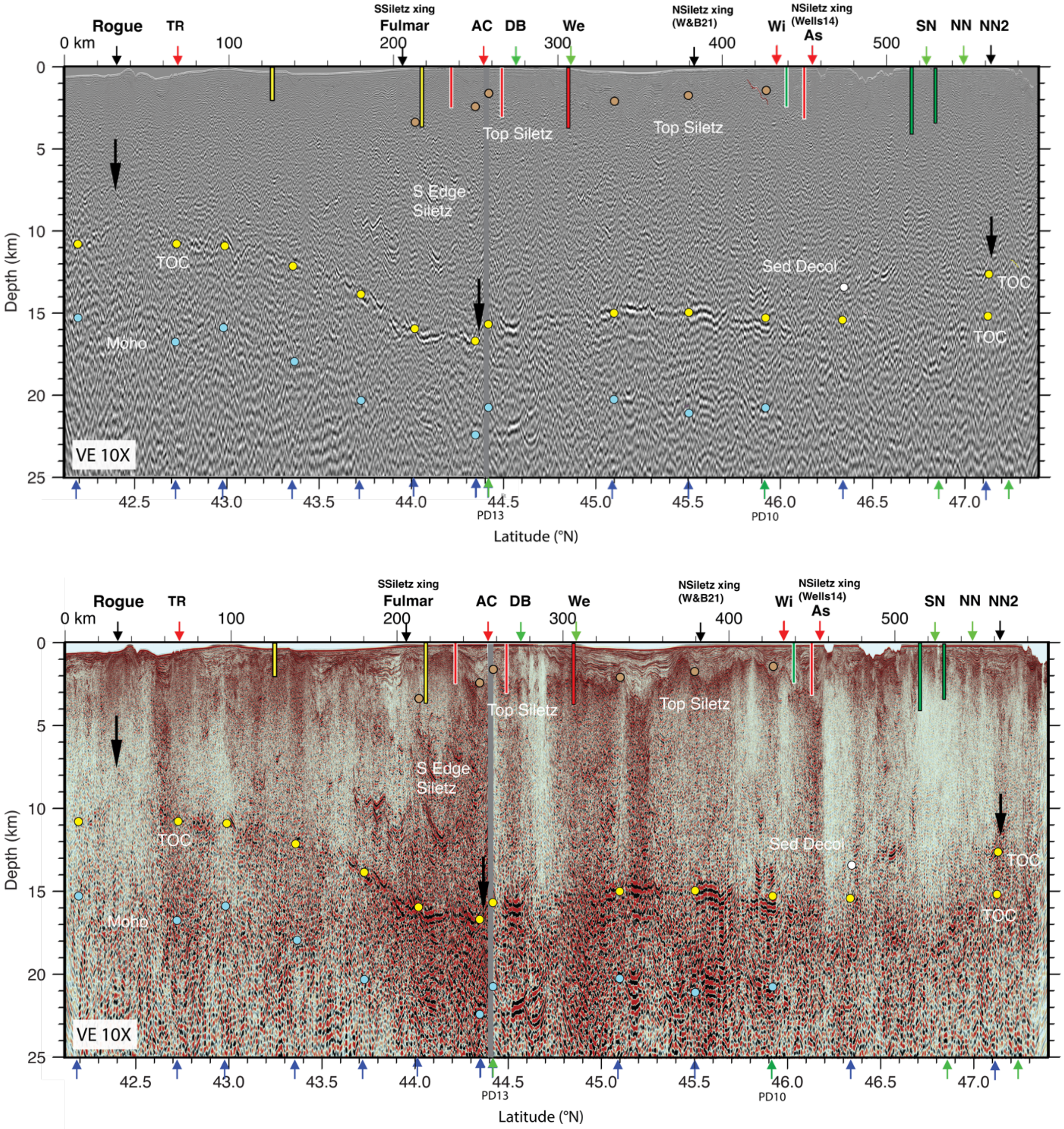

Figure S5. Along shelf seismic transect PS01 shown without horizon interpretations and displayed with and without AGC. Top panel corresponds to image in Fig. 4 shown in black-white color scheme. Bottom panel is same section without AGC applied and displayed in red-white-black color scheme. Horizon depths at line crossings are indicated with colored circles and colored arrows point to horizons interpreted in Fig. 4. Other annotations and labels as in Fig. 4.

Note that seismic reflectivity in the region of the interpreted NN2 fault is complex with ambiguous TOC and Moho horizons evident in the seismic images. The step up in TOC across the NN2 fault is identified primarily based on the shift in the depth of the band of higher reflectivity interpreted

to mark the oceanic crust, which south of 46.5°N is bounded by clear bright TOC and Moho reflections and which is best illustrated in the seismic image without AGC. The weak discontinuous events interpreted as TOC from ~46.25-47.8°N are from the top of this band of reflectivity. Step-up in basement depth at the projected NN2 fault is also required by TOC depths that are well defined on the other dip and strike lines in this region and is supported by other prior seismic imaging which indicates a several kilometer difference in plate depth under the shelf north and south of 47°N (79).

Note mismatch in Moho depth at projected PD13 line crossing (line terminates within data gap in PS01 ~2 km to west). This mismatch could reflect imaging artifacts due to the break in PS01 transect at this location or could reflect crustal thickening associated with nearby seamounts inferred in (48).

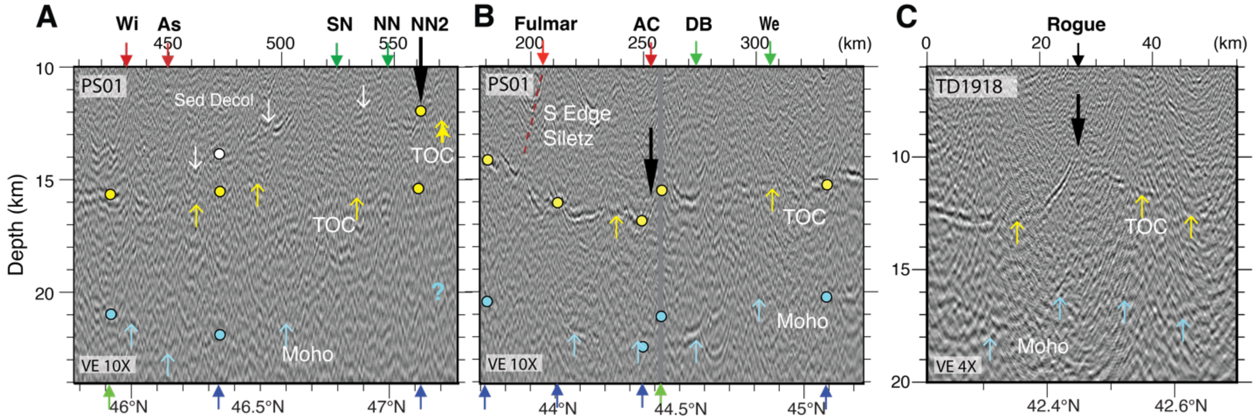

Figure S6. Seismic images from Figure 5 shown without horizon interpretations. Horizon depths at line crossings are indicated with colored circles and colored arrows point to some of the interpreted horizons from Fig. 5. Other annotations as in Figure 4.

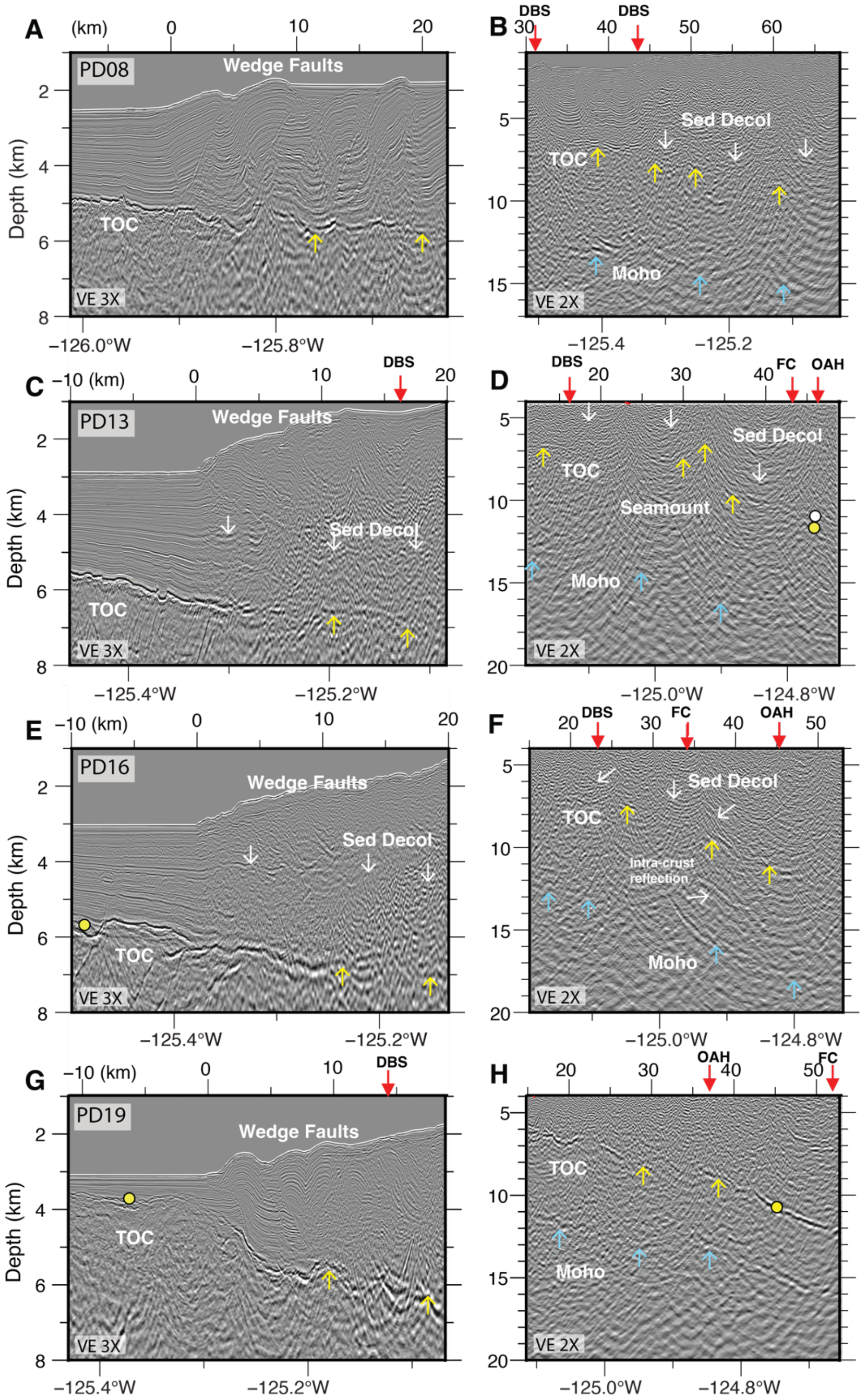

Figure S7. Seismic images from Figure 6 shown without horizon interpretations except for primary wedge faults which are shown in thin red line to aid recognition of sediment décollement horizon. Horizon depths at line crossings are indicated with colored circles and colored arrows point to horizons interpreted in Fig. 6. Annotations as in Figure 6.

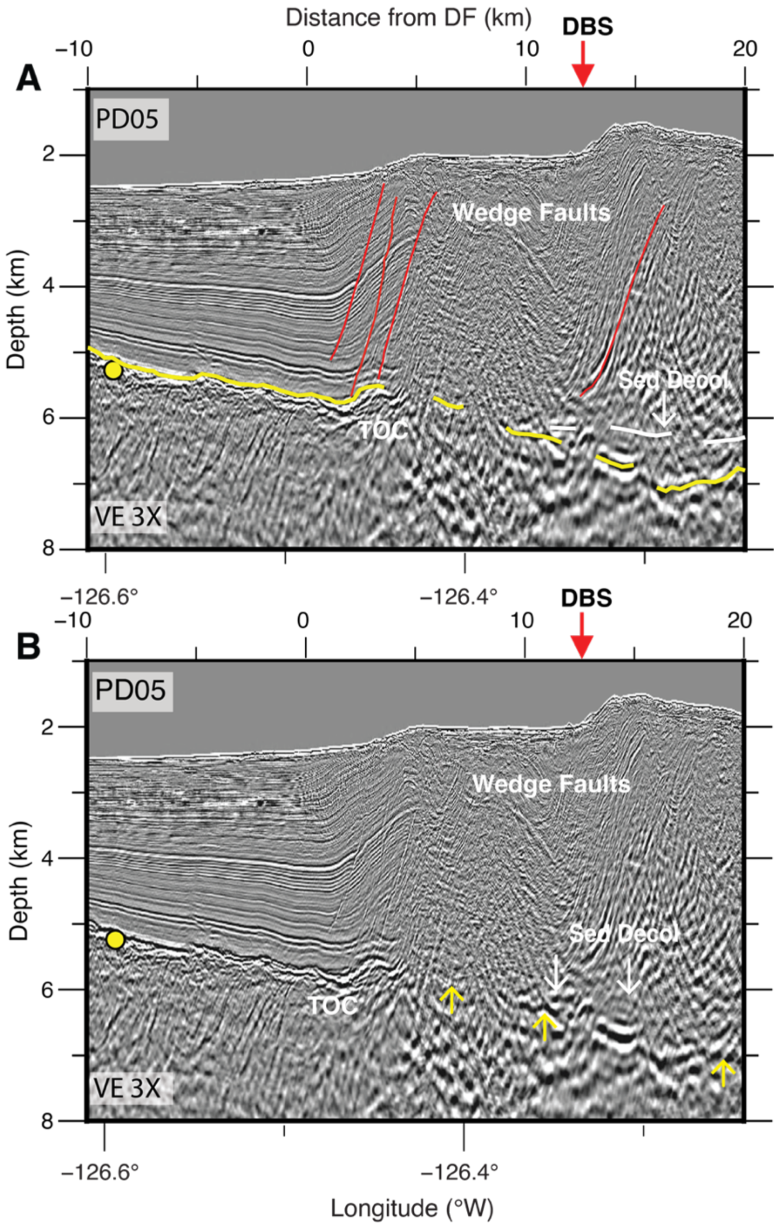

Figure S8. Close up of portion of seismic transect PD05 offshore Vancouver Island showing frontal thrusts reaching to TOC. Further downdip they shoal to a shallower horizon interpreted as plate interface décollement which merges with TOC at ~ 10 km from DF. Note change in TOC reflectivity character below sediment décollement as seen in S8. **(A)** With interpretations. **(B)** Without interpretations. Horizon depths at line crossings are indicated with colored circles and colored arrows point to some of the reflections interpreted in panel **A**.

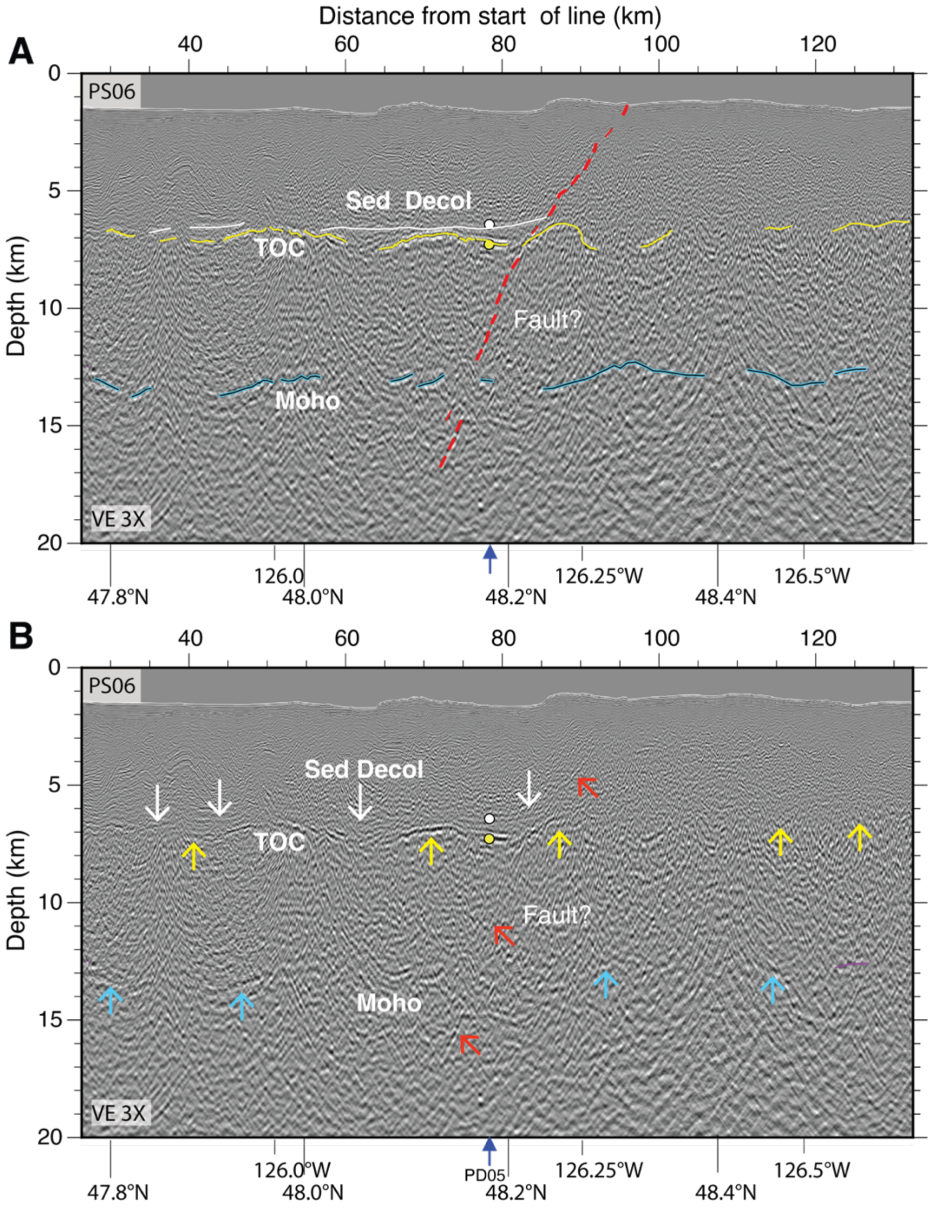

Figure S9. Seismic image of portion of seismic transect PS06 offshore Vancouver Island showing sediment décollement interpreted to extend through part of this region to ~48.25°N (km 85). TOC beneath sediment décollement is a brighter and lower frequency (wider in depth section) event compared with where sediment décollement is not identified, suggesting presence of a package of subducting lower Vp and higher attenuation (perhaps fluid rich) sediment above. The sediment décollement terminates at a likely fault bounded basement high with fault plane reflections evident which transect the oceanic crust extending into the mantle. Within the sediment column above this lower plate fault, a fault plane reflection extends through the sediment section. **(A)** With interpretations. **(B)** Without interpretations. Horizon depths at line crossings are indicated with colored circles and colored arrows point to reflections interpreted in panel **A**.

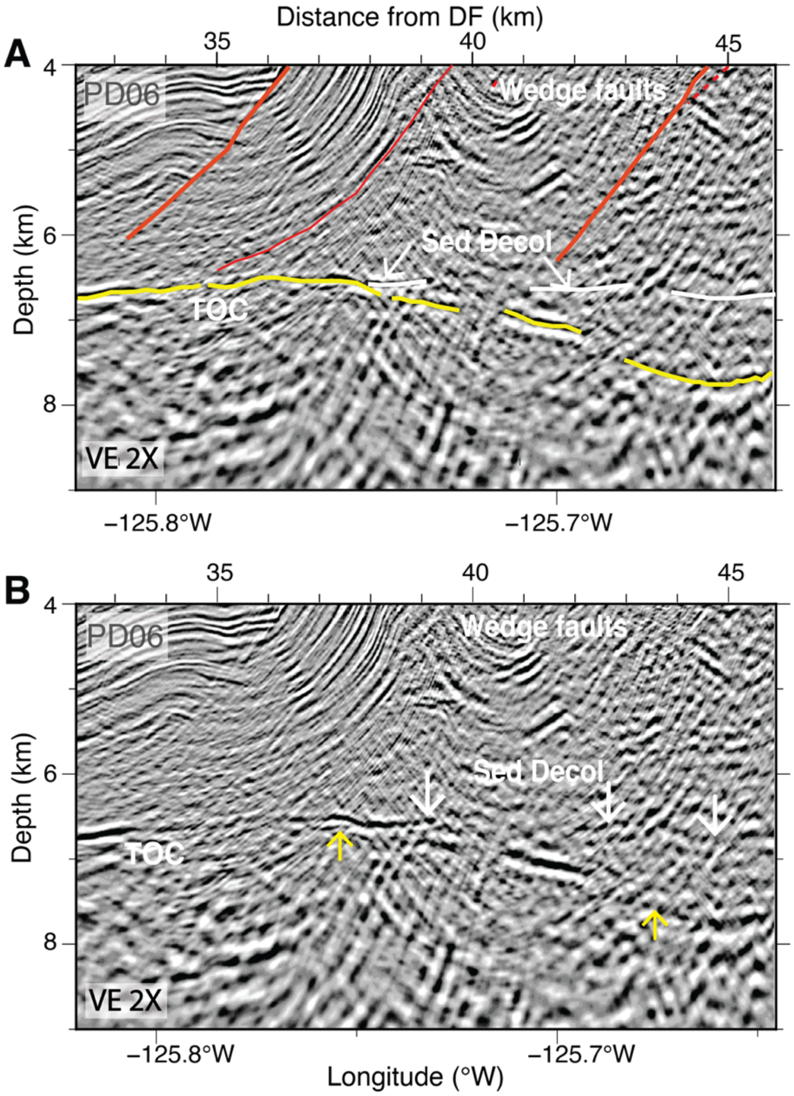

Figure S10. Close up of portion of seismic transect PD06 offshore Washington showing horizon interpreted as plate interface fault within deep sediments merging with top crust at ~ 38 km from DF. Note change in TOC reflectivity character below sediment décollement as seen in S8 and S9. **(A)** With interpretations. **(B)** Without interpretations. Horizon depths at line crossings are indicated with colored circles.

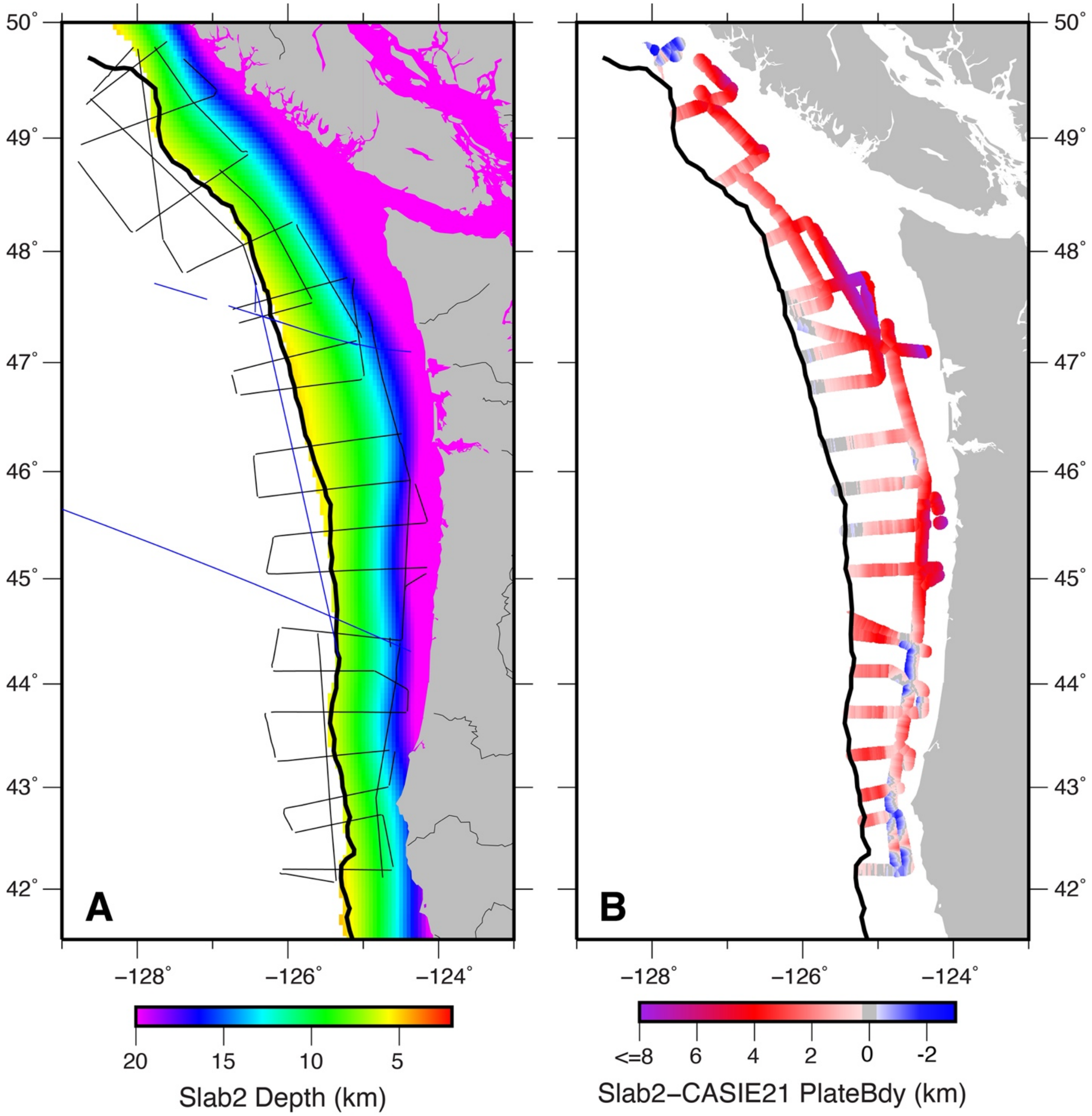

Figure S11. Comparison of new plate interface geometry and Slab2 regional plate model (27). **(A)** Slab depth from Slab2 with tracks lines for current study superimposed. **(B)** Difference between Slab2 model and plate interface depths from new study also shown in Figure 8. Positive values (red to purple) indicate new plate model is shallower than Slab2; negative values (blues) indicate new model is deeper.

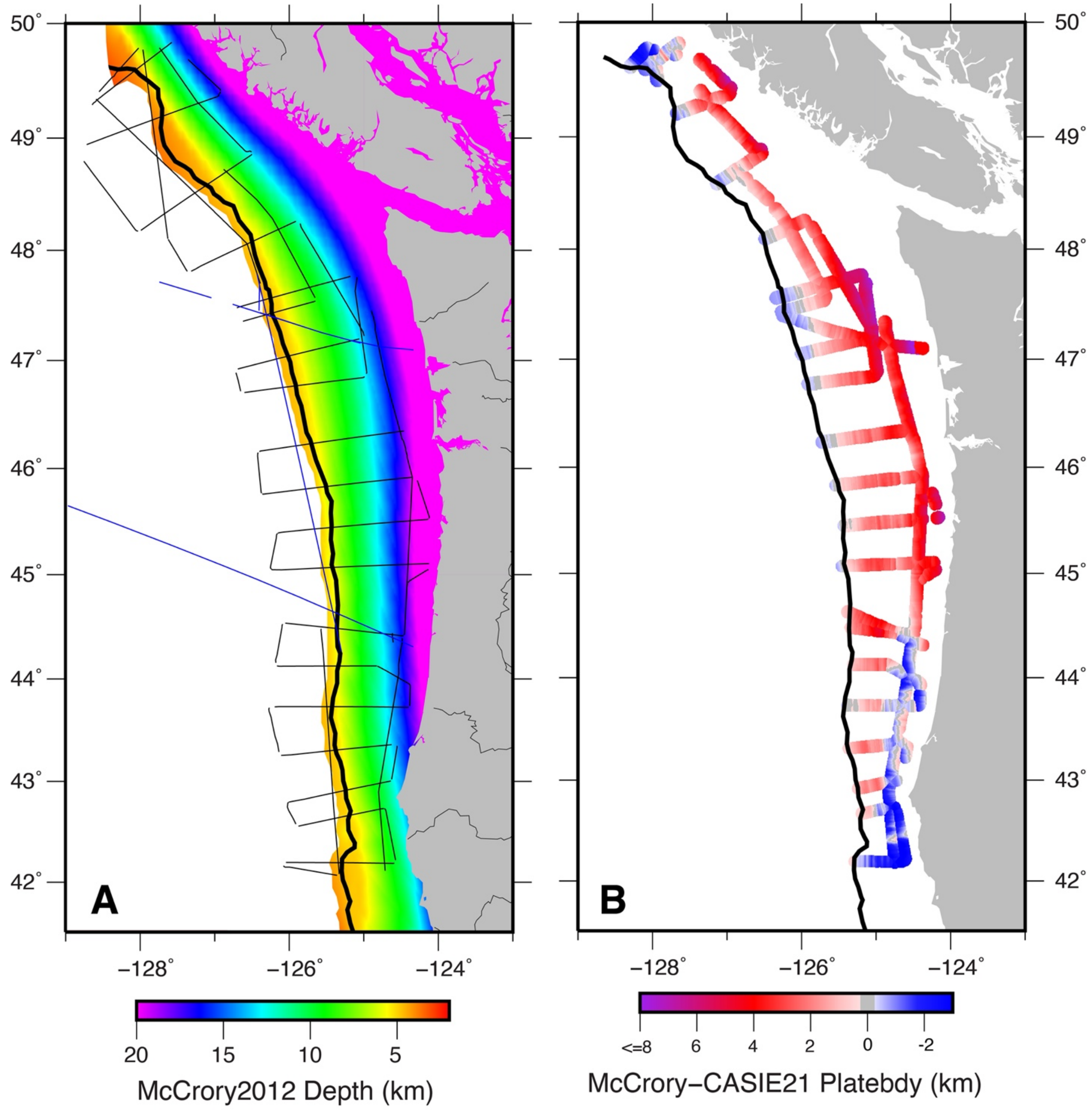

Figure S12. Comparison of new plate interface geometry and prior regional plate model of (24). **(A)** McCrory Slab depth from (24) with tracks lines for current study superimposed. **(B)** Difference between McCrory model and plate interface depths from new study. Positive values (red to purple) indicate new plate model is shallower than McCrory model; negative values (blues) indicate new model is deeper.

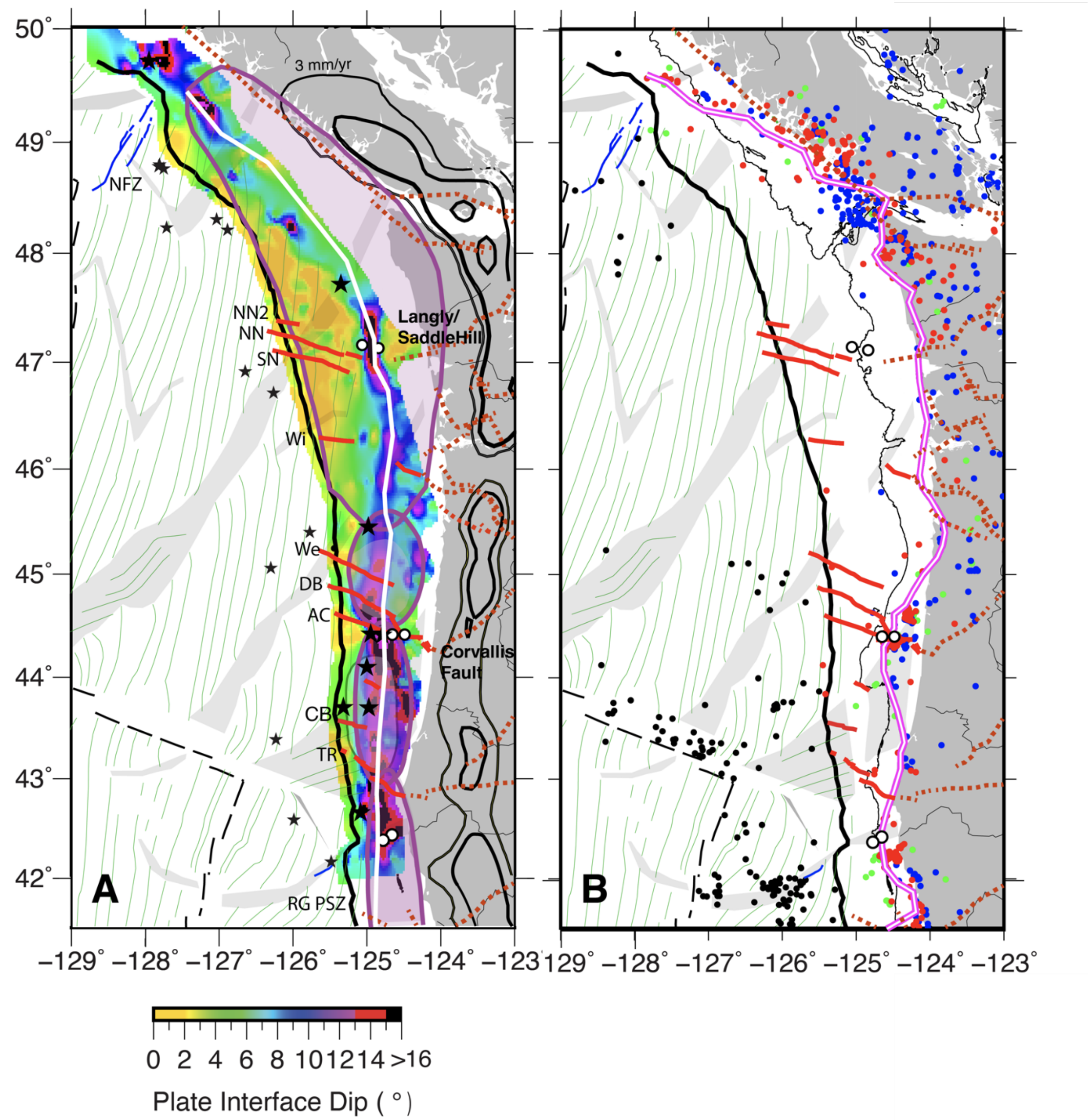

Figure S13. Comparison of geometry of plate interface with additional rupture history and slip behavior indicators. **(A)** Dip of plate interface from Fig 7B and 9 with additional features including the maximum extent of modelled slip patches for the 1700 CE earthquake of (22) in purple semi-transparent polygons; location of Outer Arc High from (17) in white. **(B)** Seismicity catalog from (20) for comparison. Seismicity is classified as from plate interface (red), lower plate (green), upper plate (blue), and incoming plate (black) using Slab2 depth model. Purple/white line shows the interpreted downdip extent of the seismogenic zone based on detected plate interface microseismicity. Thin black line corresponds with 200m bathymetric contour roughly defining the shelf edge. Other annotations as in Figure 7B.

REFERENCES AND NOTES

1. T. Lay, H. Kanamori, L. Ruff, The asperity model and the nature of large subduction zone earthquakes. *Earthq. Predict. Res.* **1**, 3–71 (1982).

2. T. Lay, S. P. Nishenko, Updated concepts of seismic gaps and asperities to assess great earthquake hazard along South America. *Proc. Natl. Acad. Sci. U.S.A.* **119**, e2216843119 (2022).

3. M. A. L. Walton, L. M. Staisch, T. Dura, J. K. Pearl, B. Sherrod, J. Gomberg, S. Engelhart, A. Tréhu, J. Watt, J. Perkins, R. C. Witter, N. Bartlow, C. Goldfinger, H. Kelsey, A. E. Morey, V. J. Sahakian, H. Tobin, K. Wang, R. Wells, E. Wirth, Toward an integrative geological and geophysical view of Cascadia subduction zone earthquakes. *Annu. Rev. Earth Planet. Sci.* **49**, 367–398 (2021).

4. K. Wang, A. M. Tréhu, Invited review paper: Some outstanding issues in the study of great megathrust earthquakes—The Cascadia example. *J. Geodyn.* **98**, 1–18 (2016).

5. Q. Bletery, A. M. Thomas, A. W. Rempel, L. Karlstrom, A. Sladen, L. De Barros, L., Mega-earthquakes rupture flat megathrusts. *Science* **354**, 1027–1031 (2016).

6. H. Kopp, Invited review paper: The control of subduction zone structural complexity and geometry on margin segmentation and seismicity. *Tectonophysics* **589**, 1–16 (2013).

7. E. A. Wirth, V. J. Sahakian, L. M. Wallace, D. Melnick, The occurrence and hazards of great subduction zone earthquakes. *Nat. Rev. Earth Environ* **3**, 125–140 (2022).

8. K. Wang, S. L. Bilek, Do subducting seamounts generate or stop large earthquakes? *Geology* **39**, 819–822 (2011).

9. R. D. Hyndman, K. Wang, The rupture zone of Cascadia great earthquakes from current deformation and the thermal regime. *J. Geophys. Res.* **100**, 22133–22154 (1995).

10. D. M. Saffer, H. J. Tobin, Hydrogeology and mechanics of subduction zone forearcs: Fluid flow and pore pressure. *Annu. Rev. Earth Planet. Sci.* **39**, 157–186 (2011).

11. J. Hunter, A. B. Watts, Gravity anomalies, flexure and mantle rheology seaward of circum-Pacific trenches. *Geophys. J. Int.* **207**, 288–316 (2016).

12. D. J. Shillington, A. Bécel, M. R. Nedimović, H. Kuehn, S. C. Webb, G. A. Abers, K. M. Keranen, J. Li, M. Delescluse, G. A. Mattei-Salicrup, Link between plate fabric, hydration and subduction zone seismicity in Alaska. *Nat. Geosci.* **8**, 961–964 (2015).

13. A. F. Arnulf, D. Bassett, A. J. Harding, S. Kodaira, A. Nakanishi, G. Moore, Upper-plate controls on subduction zone geometry, hydration and earthquake behaviour. *Nat. Geosci.* **15**, 143–148 (2022).

14. A. M. Tréhu, K. Davenport, C. B. Kenyon, S. M. Carbotte, J. L. Nabelek, D. R. Toomey, W. S. D. Wilcock, Deformation of the Juan de Fuca plate beneath the central Cascadia continental margin (44°-45°N) in response to an upper plate load. *Syst. Soc.* **3**, (2023).

15. D. Bassett, D. T. Sandwell, Y. Fialko, and A. B. Watts, Upper-plate controls on co-seismic slip in the 2011 magnitude 9.0 Tohoku-oki earthquake. *Nature* **531**, 92–96 (2016).

16. V. Sallares, C. R. Ranero, Upper-plate rigidity determines depth-varying rupture behaviour of megathrust earthquakes. *Nature* **576**, 96–101 (2019).

17. J. T. Watt, D. S. Brothers, Systematic characterization of morphotectonic variability along the Cascadia convergent margin: Implications for shallow megathrust behavior and tsunami hazards. *Geosphere* **17**, 95–117 (2021).

18. M. R. Brudzinski, R. M. Allen, Segmentation in episodic tremor and slip all along Cascadia. *Geology* ***35***, 907–910 (2007).

19. C. Goldfinger, C. H. Nelson, A. E. Morey, J. Joel, J. Patton, E. Karabanov, J. Gutierrez-Pastor, A. Eriksson, E. Gracia, G. Dunhill, R. Enkin, A. Dallimore, T. Valiier, Turbidite event history—Methods and implications for Holocene Paleoseismicity of the Cascadia subduction zone. *U.S. Geological Survey Professional Paper* 1661-F (2012).

20. E. A. Morton, S. L. Bilek, C. A. Rowe, Cascadia subduction zone fault heterogeneities from newly detected small magnitude earthquakes. *J. Geophys. Res.* **128**, e2023JB026607 (2023).

21. G. M. Schmalzle, R. McCaffrey, K. C. Creager, Central Cascadia subduction zone creep. *Geochem. Geophys. Geosyst.* **15**, 1515–1532 (2014).

22. P.-L. Wang, S. E. Engelhart, K. Wang, A. D. Hawkes, B. P. Horton, A. R. Nelson, R. C. Witter, Heterogeneous rupture in the great Cascadia earthquake of 1700 inferred from coastal subsidence estimates. *J. Geophys. Res.* **118**, 2460–2473 (2013).

23. R. E. Wells, R. J. Blakely, Y. Sugiyama, D. W. Scholl, P. A. Dinterman, Basin-centered asperities in great subduction zone earthquakes: A link between slip, subsidence, and subduction erosion? *J. Geophys. Res.* **108**, 2507 (2003).

24. P. A. McCrory, J. L. Blair, F. Waldhauser, D. H. Oppenheimer, Juan de Fuca slab geometry and its relation to Wadati-Benioff zone seismicity. *J. Geophys. Res.* **117**, B09306 (2012).

25. I. Stone, J. E. Vidale, S. Han, E. Roland, Catalog of offshore seismicity in Cascadia: Insights into the regional distribution of microseismicity and its relation to subduction processes. *J. Geophys. Res.* **123**, 641–652 (2018).

26. P. A. McCrory, J. L. Blair, D. H. Oppenheimer, S. R. Walter, Depth to the Juan de Fuca slab beneath the Cascadia subduction margin–A 3-D model for sorting earthquakes. *USGS Data Series 91*, Reston, VA (2004).

27. G. P. Hayes, G. L. Moore, D. E. Portner, M. Hearne, H. Flamme, M. Furtney, G. M. Smoczyk, Slab2, a comprehensive subduction zone geometry model. *Science* **362**, 58–61 (2018).

28. J. P. Canales, N. C. Miller, W. Baldwin, S. M. Carbotte, S. Han, B. Boston, H. Jian, J. Collins, D. Lizarralde, CASIE21-OBS: An Open-Access, OBS controlled-source seismic dataset for investigating the structure and properties of the Cascadia accretionary wedge and the downgoing explorer-Juan de Fuca-Gorda Plate System. *Seismological Research Letters*, (2023).

29. D. S. Wilson, The Juan de Fuca plate and slab: Isochron structure and Cenozoic plate motions, in *The Cascadia Subduction Zone and Related Subduction Systems—Seismic Structure, Intraslab Earthquakes and Processes, and Earthquake Hazards*, S. Kirby, K. Wang, S. Dunlop, Eds. pp. 9–12, U.S. Geological Survey Open-File Report 02–328 (2002).

30. C. DeMets, R. G. Gordon, D. F Argus, Geologically current plate motions. *Geophys. J. Int.* **181**, 1–80 (2010).

31. R. Wells, D. Bukry, R. Friedman, D. Pyle, R. Duncan, P. Haeussler, J. Wooden, Geologic history of Siletzia, a large igneous province in the Oregon and Washington coast range: Correlation to the geomagnetic polarity time scale and implications for a long-lived Yellowstone hotspot. *Geosphere* **10**, 692–719. (2014).

32. R. McCaffrey, R. W. King, S. J. Payne, M. Lancaster, Active tectonics of northwestern U.S. inferred from GPS-derived surface velocities. *J. Geophys. Res.* **118**, 709–723 (2013).

33. C. Goldfinger, L. D. Kulm, R. S. Yeats, C. Hummon, G. J. Huftile, A. R. Niem, C. G. Fox, L. C. McNeill, Oblique strike-slip faulting of the Cascadia submarine forearc: The Daisy Bank fault zone off central Oregon, in *Subduction Top to Bottom*, AGU Geophysical Monograph 96 (Washington, D.C., American Geophysical Union, 1996), pp. 65–74.

34. C. Goldfinger, L. D. Kulm, R. S. Yeats, L. McNeill, C. Hummon, Oblique strike-slip faulting of the Central Cascadia submarine forearc. *J. Geophys. Res.* **102**, 8217–8243 (1997).

35. R. D. Hyndman, G. D. Spence, T. Yuan, E. E. Davis, G. K. Westbrook, C. Bobb, R. J. Musgrave, Regional geophysics and structural framework of the Vancouver Island margin accretionary prism, in *Proceedings of the Ocean Drilling Program, initial reports* (College Station, Texas: Ocean Drilling Program; 1994) Vol. 146, No. Part 1, pp. 399–419.

36. A. M. Tréhu, I. Asudeh, T. M. Brocher, J. H. Luetgert, W. D. Mooney, J. L. Nabelek, Y. Nakamura, Crustal architecture of the Cascadia forearc. *Science* **266**, 237–243 (1994).

37. S. H. Clarke Jr., Geology of the Eel River basin and adjacent region: Implications for late Cenozoic tectonics of the southern Cascadia subduction zone and Mendocino triple junction. *AAPG Bull.* **76**, 199–224 (1992).

38. P. D. Snavely Jr., R. E. Wells, Cenozoic evolution of the continental margin of Oregon and Washington, *in* Assessing earthquake hazards and reducing risk in the Pacific Northwest, A. M. Rogers,

T. J. Walsh, W. J. Kockleman, G. R. Priest, Eds. U.S. Geological Survey Professional Paper 1560, p. 161–182, (1996).

39. P. D. Snavely Jr, Tertiary geologic framework, neotectonics, and petroleum potential of the Oregon-Washington continental margin, in Geology and resource potential of the continental margin of Western North America and adjacent ocean basins–Beaufort Sea to Baja California, D. W. Scholl, A. Granz, J. D. Vedder, Eds. Circum-Pacific Council for Energy and Mineral Resources Earth Science Series **6**, 305–335 (1987).

40. M. T. Brandon, M. K. Roden-Tice, J. I. Garver, Late Cenozoic exhumation of the Cascadia accretionary wedge in the Olympic Mountains, northwest Washington State. *Geol. Soc. Am. Bull.* **110**, 985–1009 (1998).

41. R. E. Wells, R. J. Blakely, A. G. Wech, P. A. McCrory, A. Michael, Cascadia subduction tremor muted by crustal faults. *Geology* **45**, 515–518 (2017).

42. G. D. Egbert, B. Yang, P. A. Bedrosian, K. Key, D. W. Livelybrooks, A. Schultz, A. Kelbert, Fluid transport and storage in the Cascadia forearc influenced by overriding plate lithology. *Nat. Geosci.* **15**, 677–682 (2022).

43. R. J. Burgette, R. J. Weldon, D. A. Schmidt, Interseismic uplift rates for western Oregon and along-strike variation in locking on the Cascadia subduction zone. *J. Geophys. Res.* **114**, B01408 (2009)

44. K. M. Rohr, K. P. Furlong, M. Riedel, Initiation of strike-slip faults, serpentinization, and methane: The Nootka fault zone, the Juan de Fuca-Explorer plate boundary. *Geochem. Geophys. Geosyst.* **19**, 4290–4312 (2018).

45. E. E. Davis, R. D. Hyndman, Accretion and recent deformation of sediments along the northern Cascadia subduction zone. *Geol. Soc. Am. Bull.* **101**, 1465–1480 (1989).

46. R. S. Crosson, T. J. Owens, Slab geometry of the Cascadia subduction zone beneath Washington from earthquake hypocenters and teleseismic converted waves. *Geophys. Res. Lett.* **14**, 824–827 (1987).

47. P. Audet, M. G. Bostock, D. C. Boyarko, M. R. Brudzinski, R. M. Allen, Slab morphology in the Cascadia fore arc and its relation to episodic tremor and slip. *J. Geophys. Res.* **115** (2010).

48. A. M. Tréhu, R. J. Blakely, M. C. Williams, Subducted seamounts and recent earthquakes beneath the central Cascadia forearc. *Geology* **40**, 103–106 (2012).

49. T. Finley, K. Morell, L. Leonard, C. Regalla, S. T. Johnston, W. Zhang, Ongoing oroclinal bending in the Cascadia forearc and its relation to concave-outboard plate margin geometry. *Geology* **47**, 155–158, (2019).

50. J. D. Chaytor, C. Goldfinger, R. P. Dziak, C. G. Fox, Active deformation of the Gorda plate: Constraining deformation models with new geophysical data. *Geology* **32**, 353–356 (2004).

51. E. R. Flueh, M. A. Fisher, J. Bialas, J. R. Childs, D. Klaeschen, N. Kukowski, T. Parsons, D. W. Scholl, U. ten Brink, A. M. Tréhu, N. Vidal, New seismic images of the Cascadia subduction zone from cruise SO108—ORWELL. *Tectonophysics* **293**, 69–84 (1998).

52. S. Han, N. L. Bangs, S. M. Carbotte, D. M. Saffer, J. C. Gibson, Links between sediment consolidation and Cascadia megathrust slip behaviour. *Nat. Geosci.* **10**, 954–959 (2017).

53. D. E. Peterson, K. M. Keranen, A high wave speed basal sedimentary layer identified from seismic imaging of the plate boundary in central Cascadia. *J. Geophys. Res.* **124**, 6832–6847 (2019).

54. G. Booth-Rea, D. Klaeschen, I. Grevemeyer, T. Reston, Heterogeneous deformation in the Cascadia convergent margin and its relation to thermal gradient (Washington, NW USA). *Tectonics* **27**, (2008).

55. A. J. Calvert, L. A. Preston, A. M. Farahbod, Sedimentary underplating at the Cascadia mantle-wedge corner revealed by seismic imaging. *Nat. Geosci.* **4**, 545–548 (2011).

56. M. E. MacKay, Structural variation and landward vergence at the toe of the Oregon accretionary prism. *Tectonics* **14**, 1309–1320 (1995).

57. T. Sun, D. Saffer, S. Ellis, Mechanical and hydrological effects of seamount subduction on megathrust stress and slip. *Nat. Geosci.* **13**, 249–255 (2020).

58. A. C. Kemp, N. Cahill, S. E. Engelhart, A. D. Hawkes, K. Wang, Revising estimates of spatially variable subsidence during the A.D. 1700 Cascadia earthquake using a Bayesian foraminiferal transfer function. *Bull. Seismol. Soc. Am.* **108**, 654–673 (2018).

59. C. Goldfinger, S. Galer, J. Beeson, T. Hamilton, B. Black, C. Romsos, J. Patton, C. H. Nelson, R. Hausmann, A. Morey, The importance of site selection, sediment supply, and hydrodynamics: A case study of submarine paleoseismology on the northern Cascadia margin, Washington USA. *Marine Geol.* **384**, 4–46 (2017).

60. N. M. Bartlow, A Long-term view of episodic tremor and slip in Cascadia. *Geophys. Res. Lett.* **47** (2020).

61. W. M. Bloch, M. Bostock, P. Audet, A Cascadia slab model from receiver functions. *Geochem. Geophys. Geosyst.* **24**, e2023GC011088 (2023).

62. S. Li, K. Wang, Y. Wang, Y. Jiang, S. E. Dosso, Geodetically inferred locking state of the Cascadia megathrust based on a viscoelastic Earth model. *J. Geophys. Res.* **123**, 8056–8072 (2018).

63. S. Han, S. M. Carbotte, J. P. Canales, M. R. Nedimović, H. Carton, J. C. Gibson, G. W. Horning, Seismic reflection imaging of the Juan de Fuca plate from ridge to trench: New constraints on the distribution of faulting and evolution of the crust prior to subduction. *J. Geophys. Res.* **121**, 1849–1872 (2016).

64. N. L. Bangs, J. Morgan, A. M. Tréhu, E. Contreras-Reyes, A. Arnulf, S. Han, K. M. Olsen, E. Zhang, Basal accretion along the South Central Chilean margin and its relationship to great earthquakes. *J. Geophys. Res.* **125**, e2020JB019861 (2020).

65. D. W. Scholl, S. H. Kirby, R. von Huene, H. Ryan, R. E. Wells, E. L. Geist, Great (>=Mw 8.0) megathrust earthquakes and the subduction of excess sediment and bathymetrically smooth seafloor. *Geosphere* **11**, 236–265 (2015).

66. R. Herrendörfer, Y. Van Dinther, T. Gerya, T. L. A. Dalguer, Earthquake supercycle in subduction zones controlled by the width of the seismogenic zone. *Nat. Geosci.* **8**, 471–474 (2015).

67. D. Melgar, Was the January 26th, 1700 Cascadia earthquake part of a rupture sequence? *J. Geophys. Res.* **126**, e2021JB021822 (2021).

68. A. M. Tréhu, J. Braunmiller, E. Davis, Seismicity of the central Cascadia continental margin near 44.5 N: A decadal view. *Seismol. Res. Lett.* **86**, 819–829 (2015).

69. B. T. Philip, E. A. Solomon, D. S. Kelley, A. M. Tréhu, T. L. Whorley, E. Roland, M. Tominaga, R. W. Collier, Fluid sources and overpressures within the central Cascadia Subduction Zone revealed by a warm, high-flux seafloor seep. *Sci. Adv.* **9**, eadd6688 (2023).

70. L. M. Staisch, M. A. Walton, Cascadia subduction zone database: Compilation of published datasets relevant to Cascadia subduction zone earthquake hazards and tectonics. U.S. Geological Survey data release (2022); https://doi.org/10.5066/P9O69X6E.

71. L. C. McNeill, C. Goldfinger, L. D. Kulm, R. S. Yeats, Tectonics of the Neogene Cascadia forearc basin: Investigations of a deformed late Miocene unconformity. *Geol. Soc. Am. Bull.* **112**, 1209–1224 (2000).

72. H. J. Tobin, J. C. Moore, M. E. Mackay, D. L. Orange, L. D. Kulm, Fluid flow along a strike-slip fault at the toe of the Oregon accretionary prism: Implications for the geometry of frontal accretion. *Geol. Soc. Am. Bull.* **105,** 569–582 (1993).

73. T. Gerya, Numerical modeling of subduction: State of the art and future directions. *Geosphere* **18**, 503–561 (2022).

74. S. Carbotte, S. Han, B. Boston, Multi-channel seismic shot data from the Cascadia margin acquired during Langseth cruise MGL2104 (2021), Marine Geoscience Data System, (2022). 10.26022/IEDA/330905.

75. W. J. Stephenson, N. G. Reitman, S. J. Angster, P- and S-wave velocity models incorporating the Cascadia subduction zone for 3D earthquake ground motion simulations, version 1.6—Update for Open-File Report 2007–1348 (ver. 1.1, Sept. 10, 2019): U.S. Geological Survey Open-File Report 2017–1152, 17 p., https://doi.org/10.3133/ofr20171152. [Supersedes USGS Open-File Report 2007–1348.] (2017).

76. G. Horning, J. P. Canales, S. M. Carbotte, S. Han, H. Carton, M. R. Nedimović, P. E. Van Keken, A 2-D tomographic model of the Juan de Fuca plate from accretion at axial seamount to subduction at the Cascadia margin from an active source ocean bottom seismometer survey. *J. Geophys. Res.* **121**, 5859–5879 (2016).

77. S. Carbotte, S. Han, B. Boston, J. Caneales, ION Geophysical team (2023). Processed pre-stack depth-migrated seismic reflection data from the CASIE21 multi-channel seismic survey (MGL2104*)*, Marine Geoscience Data System, (2023). 10.26022/IEDA/331274.

78. P. Wessel, J. F. Luis, L. Uieda, R. Scharroo, F. Wobbe, W. H. F. Smith, D. Tian, The Generic Mapping Tools version 6. *Geochem. Geophys. Geosyst.* **20**, 5556–5564. (2019).

79. M. A. Fisher, R. D. Hyndman, S. Y. Johnson, T. M. Brocher, R. S. Crosson, R. E. Wells, A. J. Calvert, U. S. tene Brink, Crustal Structure and Earthquake Hazards of the Subduction Zone in Southwestern British Columbia and Western Washington. *U.S. Geological Survey Professional Paper* 1661-C, (2005).
